# Contribution of Berry Polyphenols to the Human Metabolome

**DOI:** 10.3390/molecules24234220

**Published:** 2019-11-20

**Authors:** Preeti Chandra, Atul S. Rathore, Kristine L. Kay, Jessica L. Everhart, Peter Curtis, Britt Burton-Freeman, Aedin Cassidy, Colin D. Kay

**Affiliations:** 1 Food Bioprocessing & Nutrition Sciences, Plants for Human Health Institute, North Carolina State University, North Carolina Research Campus, 600 Laureate Way, Kannapolis, NC 28081, USA; pchandr3@ncsu.edu (P.C.); asrathor@ncsu.edu (A.S.R.); jeverha@ncsu.edu (J.L.E.); 2 Department of Nutrition, University of North Carolina at Chapel Hill Nutrition Research Institute, Kannapolis, NC 28081, USA; klkay4@ncsu.edu; 3 Department of Nutrition & Preventive Medicine, Norwich Medical School, University of East Anglia, Norwich NR4 7TJ, UK; P.Curtis@uea.ac.uk; 4 Department of Food Science and Nutrition and Center for Nutrition Research, Illinois Institute of Technology, Chicago, IL 60601, USA; bburton@iit.edu; 5 Institute for Global Food Security, Queen’s University Belfast, Belfast BT7 1NN, Northern Ireland; a.cassidy@qub.ac.uk

**Keywords:** mass spectrometry, metabolome, metabolomics, microbiome, (poly)phenol

## Abstract

Diets rich in berries provide health benefits, however, the contribution of berry phytochemicals to the human metabolome is largely unknown. The present study aimed to establish the impact of berry phytochemicals on the human metabolome. A “systematic review strategy” was utilized to characterize the phytochemical composition of the berries most commonly consumed in the USA; (poly)phenols, primarily anthocyanins, comprised the majority of reported plant secondary metabolites. A reference standard library and tandem mass spectrometry (MS/MS) quantitative metabolomics methodology were developed and applied to serum/plasma samples from a blueberry and a strawberry intervention, revealing a diversity of benzoic, cinnamic, phenylacetic, 3-(phenyl)propanoic and hippuric acids, and benzyldehydes. 3-Phenylpropanoic, 2-hydroxybenzoic, and hippuric acid were highly abundant (mean > 1 µM). Few metabolites at concentrations above 100 nM changed significantly in either intervention. Significant intervention effects (*p* < 0.05) were observed for plasma/serum 2-hydroxybenzoic acid and hippuric acid in the blueberry intervention, and for 3-methoxyphenylacetic acid and 4-hydroxyphenylacetic acid in the strawberry intervention. However, significant within-group effects for change from baseline were prevalent, suggesting that high inter-individual variability precluded significant treatment effects. Berry consumption in general appears to cause a fluctuation in the pools of small molecule metabolites already present at baseline, rather than the appearance of unique berry-derived metabolites, which likely reflects the ubiquitous nature of (poly)phenols in the background diet.

## 1. Introduction

There are many dietary sources of anthocyanins, with berries being the richest source. Notable amounts also come from natural colorants and red/purple varieties of grapes, plums, apples, and cabbage [1,2]. Diets rich in berries and anthocyanins per se have been shown to reduce the risk of developing cardio-metabolic disorders, reduce inflammatory status, and support gut health [1,2,3,4,5]; consequently, their intake is widely advocated. However, without appropriate characterization of the berry metabolome, along with its contribution to the human metabolome, it is difficult to accurately determine the impact of anthocyanin consumption on human physiology. 

Metabolomics is a potentially powerful tool in establishing anthocyanins’ impact on metabolic and physiological processes. However, progress has been tempered by a primary focus on endogenous nutrient metabolites, with little consideration for the potentially critical, yet largely unresolved contribution of plant/dietary phytochemicals to the human metabolome. Furthermore, the contribution of the berry metabolome, a primary dietary source of anthocyanins, to the human metabolome has yet to be established. 

The extent of (poly)phenol absorption and metabolism is underrepresented in previous research, which has focused primarily on characterizing phase II metabolites of precursor compounds (e.g., cyanidin-3-*O*-glucuronide or cyanidin-3-sulfate). Recent evidence indicates microbial-derived anthocyanin metabolites make up the majority (number and concentration) of absorbed (poly)phenols from berries [1,6], though the contribution of other phytochemicals such as flavonoids, procyanidins, elligatannins, gallotannins, and phenolic acids, which often have analogous microbial metabolites, should be considered [1]. Likewise, the contribution of the berry metabolome to the human metabolome may also be obscured by the high concentration of (poly)phenols, including flavonoids and chlorogenic acids derived from other dietary sources, for example, coffee, tea, chocolate, and wine. A high intake of the aforementioned foods could conceivably deliver 2 g/d of these precursor phytochemicals [7,8,9], which would be catabolized by the microbiome, forming identical or structurally similar absorbable metabolites to those derived from berries. 

Microbial metabolites of anthocyanins are reported to be identical to many endogenous human metabolites, such as the catecholamine metabolite DOPAC (3,4-dihydroxyphenylacetic acid), or mimic pharmacologically active agents such as the anti-inflammatory salicylic acid (2-hydroxybenzoic acid) or vasoactive acetovanillone (1-(4-hydroxy-3-methoxyphenyl)ethan-1-one). As these metabolites of dietary origin have the potential to affect human metabolic, immune, and physiological processes [1,5], they are likely to confound study findings in nutrition, pharmacology, or medicine. Moreover, microbial metabolites of anthocyanins, such as 3,4-dihydroxybenzoic acid (protocatechuic acid), 4-hydroxy-3-methoxybenzoic acid (vanillic acid), 4-hydroxy-3,5-dimethoxybenzoic acid (syringic acid), and 3,4,5-trihydroxybenzoic acid (gallic acid), are associated with beneficial changes in biomarkers of atherosclerosis, inflammation, and endothelial dysfunction in in vitro models [10,11,12,13,14]. Deeper evaluation of these compounds, and possibly others, warrants attention. As phenolic metabolites of dietary origin have been overlooked in many “traditional” metabolomics studies, reanalyzing banked tissue or databases with a focus on phytochemical metabolites of dietary origin could be invaluable in uncovering essential bioactives for health maintenance and protection from impairments in physiology.

The aim of this work was to design a comprehensive method to characterize the berry metabolome, representing a distinctive dietary category of phytochemicals, and to determine its contribution to the human metabolome in nutrition intervention studies and, ultimately, larger population-based cohort studies. We utilized a “systematic review approach/strategy” to characterize the phytochemical composition of berries, with a focus on blueberry and strawberry, as the most consumed berries in the USA [8]. The data were used to develop a tandem mass spectrometry (MS/MS), MS^n^ quantitative metabolomics methodology. A systematic review approach was necessary as the phytochemical complexity of berries is often underrepresented in food composition databases such as Phenol-Explorer (Phenol-Explorer 3.6. Retrieved September 3, 2019, from http://phenol-explorer.eu/), the US Department of Agriculture (USDA) database (USDA 3.0 Retrieved Feb 16, 2019, from https://data.nal.usda.gov/) [7], and *FoodDB* (FooDB 1.0. Retrieved September 3, 2019, from http://Foodb.ca/) [15]. These databases generally report the most abundant phytochemicals (with the exception of Foodb [15]), which, in the case of blueberry and strawberry, are anthocyanins and hydroxycinnamic acids. However, many publications also report variable concentrations of flavan-3-ols, flavanones, flavones, flavonols, phenolic acids, stilbenes, and so on, in these berries, which can contribute to the pool of human and absorbable microbial metabolites. The methodology and workflow was utilized for a secondary analysis of clinical samples (NCT02035592 [4] and NCT02612090, unpublished) exploring the contribution of phytochemicals to the human metabolome, which was not a focus of the initial studies. As there are no metabolomics methodologies or databases that fully capture the contribution of dietary phytochemicals to the human metabolome, the present workflow describes a potential first step in this direction.

## 2. Materials and Methods

Literature review. The data-capture workflow utilized a systematic review approach, using PubMed, Web of Science, and SCOPUS (Table 1), which aimed to capture composition data relative to the blueberry and strawberry metabolomes (final search on Aug 13, 2018). Exclusion criteria included the following: articles not translated into English, titles without keywords for berry-related foods, varietals not grown or consumed in the USA, berry extracts being in either a proprietary formulation or in a form not consumed by humans (e.g., stems or other agricultural by-products, or given as topical skin treatment), and animal studies (Figure S1).

**Figure 1:**
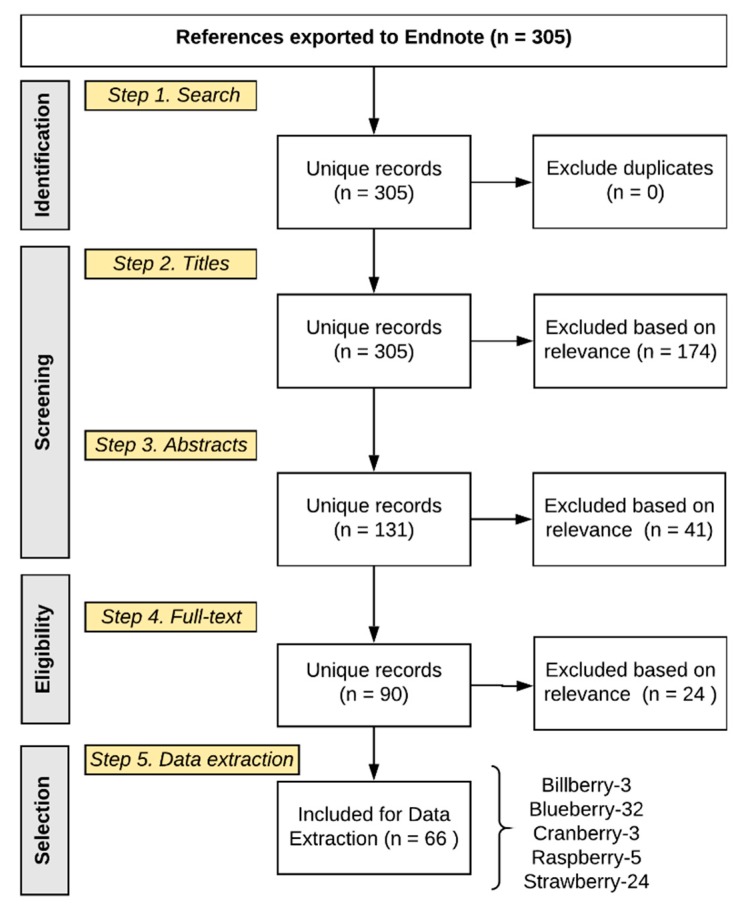
Summary of selected studies.

Abstract and full text inclusion criteria included the following: species/cultivars/varieties commonly consumed in the USA; commonly consumed food matrices/format, including whole food, juice, or minimally processed extract; and having composition data for the food or metabolites identified in human tissues. The final full text extraction of composition data was established for 90 articles meeting the inclusion criteria (Figure 1).

**Figure 2:**
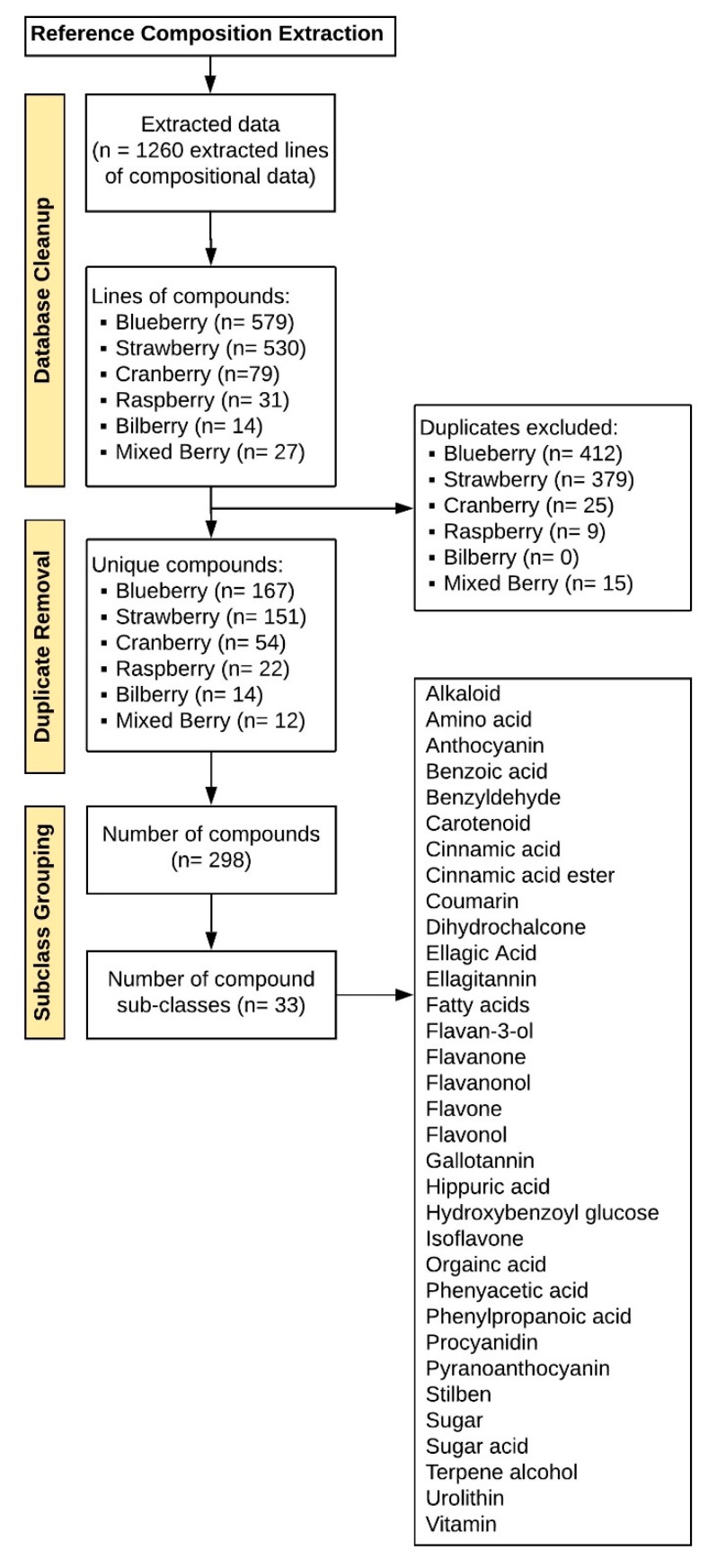
Compositional data extraction.

Data extraction revealed 298 unique compounds (Figure 2), reflecting 33 general compound classes. The major classes of berry phytochemicals were as follows: anthocyanins, flavones, flavonols, ellagitannins, procyanidins, hydroxycinnamic acids, gallotannins, sugars, hydroxybenzoic acids, and flavan-3-ols. The major classes of human metabolites (derived from either the human or gut microbiome) were anthocyanins, hydroxybenzoic acids, hydroxycinnamic acids, hydroxyphenylacetic acids, (hydroxyphenyl)propionic acids, and hippuric acids. After target identification, 98 commercially available and synthetic reference standards were obtained (Table S2) from Alfa Aesar (Tewksbury, MA, USA), Arcos Organics, (Geel, Belgium), Ark Pharm (Libertyville, IL, USA), Biovision (San Francisco, CA, USA), Extrasynthase SA (ZI Lyon Nord, France), Matrix Scientific (Columbia, SC, USA), Oxchem (Wood Dale, IL, USA), Sigma (St. Louis, MO, USA), TCI America (Portland, Oregon, USA), and Toronto Research Chemicals (Toronto, Canada), or were synthesized in a project sponsored by the BBSRC (U.K. Biotechnology and Biological Sciences Research Council; BB/H004726).

MS optimization. The analytical method optimization workflow included fragment transition optimization via direct syringe infusion of pure reference standards, following source and chromatography optimization using complex analyte mixtures. Sequential optimization of scanning and integration parameters was established in multiple reaction monitoring (MRM), scheduled MRM (sMRM), and advanced scheduled MRM (ADsMRM) modes. A mixture of reference standards was spiked in pooled extracted blank commercial human serum/plasma and injected multiple times at concentrations reflecting the lower limits of quantification (LLOQ), middle quality control (MCQ), and upper limit of quantitation (ULOQ). This process established chromatography drift, signal stability, background noise, and integration thresholds. The final method contained 142 analytes comprising 98 reference standards and 44 metabolites, for which no reference standards exist commercially (Table S2). The latter 44 metabolites, which were not commercially available, were optimized using MRM scanning mode, where transitions were programmed for their optimized precursor base structures (i.e., unconjugated/pre-metabolized analyte) and known biological conjugate masses, such as glucuronide, sulfate, glycine, and methyl. The MRMs were run on pooled samples as a series of dilutions to identify the linearity of signals (i.e., signals proportionally affected by overlapping precursor and product signals). Identification was based on fragmentation profiling of the precursor structure and 3–5 product transitions. The method was merged with the previous ADsMRM method, and utilized to interrogate 690 biospecimens, derived from studies feeding blueberry [4] and strawberry (Table 2). 

Analytical procedure. The metabolites were purified from 100 μL human plasma by 96-well plate solid phase extraction (SPE; Strata™-X Polymeric Reversed Phase, microelution 2 mg/well) [16,17]. The solid phase extracted samples were chromatographically separated and quantified using Exion ultra-high performance liquid chromatography-tandem mass spectrometry (UHPLC-ESI-MS/MS) on a SCIEX QTRAP 6500^+^ enhanced hybrid triple quadrupole-linear ion trap mass spectrometer with electrospray IonDrive Turbo-V Source. The samples were injected into a Kinetex PFP UPLC column (1.7 µm particle size, 100 Å pore size, 100 mm length, 2.1 mm internal diameter; Phenomenex^®^) with oven temperature maintained at 37 °C. Mobile phase A consisted of 0.1% *v*/*v*. formic acid in water (Optima grade, Fisher Scientific) and mobile phase B consisted of 0.1% *v*/*v*. formic acid in LC-MS grade acetonitrile (Honeywell Burdick and Jackson, Muskegon, MI, USA), with a binary gradient ranging from 2 to 90% B over 30 min and flow rate gradient from 0.55 to 0.75 mL/min. MS/MS scanning was accomplished by ADsMRM using polarity switching between positive and negative ionization mode in Analyst (v.1.6.3, SCIEX) and with quantitation conducted using MultiQuant (v.3.0.2, SCIEX) software platforms. Internal standards included l-tyrosine-^13^C_9_,^15^N, resveratrol-(4-hydroxyphenyl-^13^C_6_), and phlorizin dehydrate (Sigma-Aldrich), and for the 98 reference standards, 11-to-14-point calibration curves (1 nM to 100 μM) were utilized. Calibration curves were made by spiking reference standards in matrix matched SPE extracted human commercial serum (pooled from male AB plasma; H4522, Sigma-Aldrich Corporation, St. Louis, MO, USA). Metabolites that were not commercially available were quantified using reference standards having comparable ionization intensities and similar molecular structure (Table S2). Finally, all metabolites were verified based on established retention times (using authentic and synthesized standards where possible) and 1-to-6 qualitative transitions. Source parameters included the following: curtain gas 35, ion-spray voltage 4000, temperature 550, nebulizer gas 70, and heater gas 70. The optimized analyte specific quadrupole voltages (mean ± SD) for negative mode were 43 ± 25 for declustering potential, 10 ± 1 for entrance potential, 26 ± 11 for collision energy, and 14 ± 9 for collision cell exit potential. Similarly, for the positive mode, the optimized analyte specific quadrupole voltages (mean ± SD) were 35 ± 17 for declustering potential, 10 ± 1 for entrance potential, 20 ± 7 for collision energy, and 12 ± 11 for collision cell exit potential. 

Intervention study design. The metabolome dataset was established following secondary analysis of biological samples from two human nutrition interventions. Analyses were performed on “banked” fasting plasma/serum samples. Briefly, the blueberry intervention was a six-month double-blind randomized controlled parallel feeding study, feeding the equivalent of ½ cup or 1 cup of blueberries (fresh weight equivalent of 75 and 150 g, respectively) per day as freeze-dried powder (13 and 26 g powder, respectively) to individuals with metabolic syndrome (NCT02035592) [4]. Group differences were established using linear mixed model using STATA (Stata Corp). The strawberry intervention was a randomized double-blind crossover controlled trial feeding the equivalent of ~3.5 cup of strawberries (50 g as freeze-dried powder) per day to participants with moderately elevated plasma glucose and low density lipoprotein cholesterol (LDL) for four weeks (NCT02612090, unpublished). Intervention effects were established by comparison of four-week post-treatment concentrations (treatment vs. control; paired t-test), where baseline values were not statistically different.

## 3. Results

**Figure 3:**
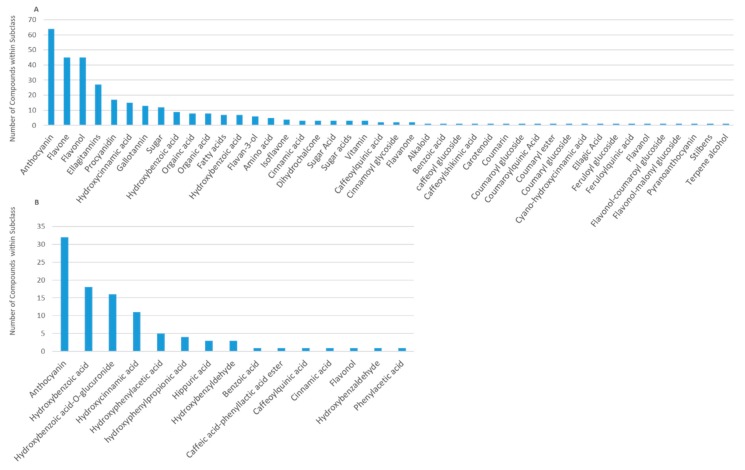
Diversity of plant secondary metabolite reported in berry studies (**A**) and their human metabolites (**B**).

Literature extraction. The literature search returned 305 results (Figure 1); 174 titles (TEXT S3, references) and 41 abstracts (TEXT S4, references) failed to meet the inclusion criteria, leaving 90 articles for full text extraction of composition data; 24 of the full text manuscripts failed to meet the final inclusion criteria (TEXT S5, references). Extracted composition information from the 66 remaining manuscripts (TEXT S6, references) provided 1260 rows of data in Excel, each reflecting a compound reported in an individual manuscript, as well as its source/matrix (food or biological tissue), quantity, concentration, and treatment serving/dose (if provided) (Figure 2). Subsequently, 840 rows of data were identified, which comprised duplicate chemical/compound names across the berries and any synonyms and/or inaccurate nomenclature. After these extraneous rows were removed (i.e., duplicate compound names, synonyms and inaccurate nomenclature), the final metabolome dataset contained 420 rows of compounds (phytochemicals or metabolites), which were reported in either studies characterizing the berry, or human tissue from nutrition intervention studies feeding berries. The final dataset consisted of 298 unique compounds after excluding duplicate compounds across berry varieties, and reflected 33 general subclasses (Figure 2). The major subclasses of phytochemicals in berries were anthocyanins, flavones, flavonols, ellagitannins, procyanidins, hydroxycinnamic acids, gallotannins, sugars, hydroxybenzoic acids, and flavan-3-ols; the major classes of human (human/microbial) metabolites were anthocyanins, hydroxybenzoic acids, hydroxycinnamic acids, hydroxyphenylacetic acids, 3-(hydroxyphenyl)propionic acids, and hippuric acids (Figure 3). 

Berry metabolome. The largest and most diverse (poly)phenol/compound subclasses reported in the literature were the anthocyanins, reflecting 64 structural derivatives, followed by flavones and flavonols (n = 45), ellagitannins (n = 27 each), procyanidins (n = 17), hydroxycinnamic acids (n = 15), gallotannins (n = 13), and hydroxybenzoic acids (n = 9), (Figure 3A). The (poly)phenols comprised the majority (in number) of reported plant secondary metabolites. 

**Figure 4:**
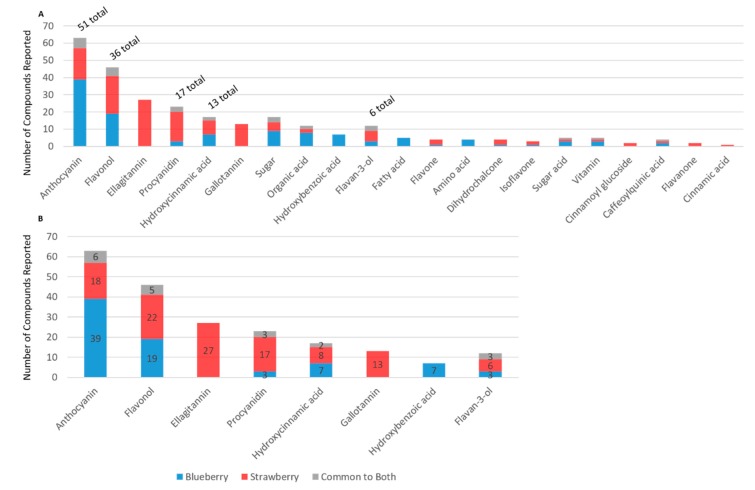
Distribution of secondary plant metabolites reported in the blueberry and strawberry. (**A**) Number of unique compounds reported in blueberry, strawberry, or common to both; (**B**) number of secondary plant metabolites/(poly)phenols reported in the most diverse (in number) subclasses.

When compositional data were available for multiple berries (beyond strawberry and blueberry), the data were extracted to explore unique or overlapping compounds across berries. The search strategy revealed only limited data for berries other than blueberry and strawberry (Figure 1). However, as expected, the available data indicated that blueberries, strawberries, and cranberries differ considerably in phytochemical compositions, particularly among anthocyanins, cinnamic acids, flavan-3-ols, flavones, flavonols, ellagitannins, and gallotannins. The present study, however, focused on blueberries and strawberries, as they are the main berries captured in epidemiological studies to date (via food frequency questionnaires), and have the most clinical trial data available. For the blueberry and strawberry data (derived from the literature), unique compounds were identified within each phytochemical subclass (Figure 4A), where there was generally less than 40% overlap in secondary plant metabolites (Figure 4B). Flavanones, ellagitannins, and gallotannins and cinnamoyl glycosides were unique to the strawberry.

**Figure 5:**
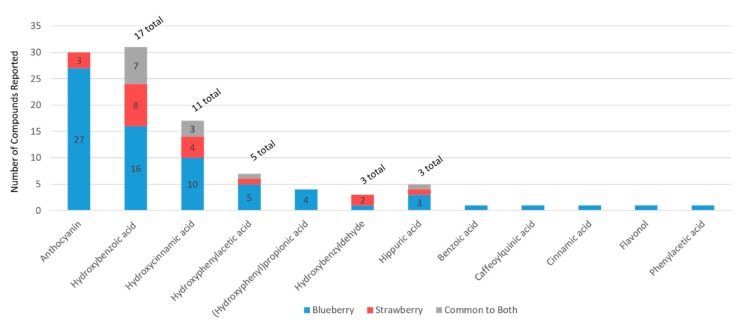
Number of human metabolites reported in intervention studies feeding blueberry or strawberry.

Human metabolome. The human metabolite diversity reported in the literature was extensive (Figure 3B), primarily reflecting smaller subclasses of microbial phenolic catabolites and having very little overlap (generally <25%) among structures within each subclass (Figure 5). The most diverse subclasses of metabolites included anthocyanins, hydroxybenzoic acids, hydroxycinnamic acids, hydroxyphenylacetic acids, 3-(hydroxyphenyl)propionic acids, hippuric acids and benzyldehydes.

**Figure 6:**
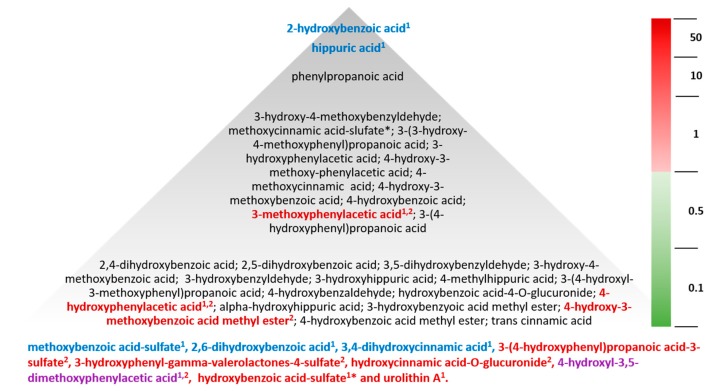
Abundance of human metabolites following consumption of blueberries and strawberries. Values reflect maximum concentration reported in a single serum/plasma sample within the blueberry [4] or strawberry intervention dataset. Bold, metabolites found to change significantly; blue, metabolites changing significantly (*p* < 0.05) following feeding with blueberry; red, metabolites changing significantly (*p* < 0.05) following feeding with strawberry; purple, metabolites changing significantly (*p* < 0.05) following feeding with blueberry or strawberry. Superscripts: ^1^ significant treatment/intervention effect; ^2^ significant within treatment group effect for change relative to baseline concentration; ^*^unknown isomeric configuration as a result of co-eluting peaks or lack of reference standard.

In the analysis of plasma/serum from the blueberry and strawberry interventions, anthocyanins were not identified in fasting samples, while the phenolic metabolites were. The metabolomes observed displayed a similar diversity of (poly)phenolic microbial metabolite subclasses (such as benzoic, cinnamic, phenylacetic, 3-(phenyl)propanoic, and hippuric acids). In some individuals, the metabolite concentrations (maximum observed concentration) were as high as 10–25 µM for a few compounds, namely 3-phenylpropanoic acid, 2-hydroxybenzoic acid, and hippuric acid; more consistently, compounds were observed between 50 and 250 nM (Figure 6) with considerable variability in concentration across participants (SD ± 400–1500 nM). Mean concentrations of individual classes of metabolites, such as benzoic, phenylacetic, 3-(phenyl)propanoic, and cinnamic acids, often reached 30–40 µM cumulatively, with their individual concentrations ranging between 10 and 100 nM.

The metabolites observed in highest abundance (maximum concentration) were consistent across the two intervention studies. The most abundant metabolites in individuals in both the strawberry and blueberry intervention were hippuric acid, reported between 10 and 20 µM, and 2-hydroxybenzoic acid, reported between 25 and 30 µM, respectively. Of these metabolites, the change in concentration was only significant (*p* < 0.05; treatment effect) for hippuric (mean change of 2.5 µM) and 2-hydroxybenzoic acid (mean change of 15 nM) in the blueberry intervention study (Figure 6) [4]. Other high abundance metabolites (maximum identified concentrations) identified in the strawberry and blueberry interventions included 3-hydroxy-4-methoxybenzyldehyde (0.8–11.7 µM), 4-methoxycinnamic acid (0.4–11.7 µM), methoxycinnamic acid sulfate (0.06–1.6 µM), 4-hydroxy-3-methoxybenzoic acid (0.3–5.8 µM), 4-hydroxybenzoic acid (0.3–1.2 µM), 3-hydroxyphenylacetic acid (0.4–1.0 µM), 4-hydroxy-3-methoxyphenylacetic acid (0.5–1.5 µM), 3-(3-hydroxy-4-methoxyphenyl)propanoic acid (0.3–1.2 µM), 3-methoxyphenylacetic acid (0.09–1.1 µM), and 3-(4-hydroxyphenyl)propanoic acid (0.6–0.8 µM) (Figure 6). However, only 3-methoxyphenylacetic acid was observed to increase significantly (*p* = 0.002) from baseline values (mean increase of 50 nM) following intervention with strawberries. Further, benzoic, cinnamic, hippuric, phenylacetic, and 3-(hydroxyphenyl)propanoic acids and benzyldehydes were observed having maximum concentrations generally below 500 nM. Of these metabolites, a significant intervention effect (57 nM mean difference) was observed for 4-hydroxyphenylacetic acid (*p* = 0.002) and a significant change from baseline was observed for 4-hydroxy-3-methoxybenzoic acid methyl ester (4.5 nM mean increase; *p* = 0.01) following the strawberry intervention only (Figure 6). However, the absolute change in concentrations was relatively small.

Treatment effects were observed for low concentration metabolites, found between 10 and 25 nM, in the blueberry intervention, including 4-methoxybenzoic acid-3-sulfate, 2,6-dihydroxybenzoic acid, and 3,4-dihydroxycinnamic acid (*p* < 0.05). Likewise, in the strawberry intervention, significant intervention differences were identified for hydroxybenzoic acid-sulfate^1^ and urolithin A, while significant changes from baseline (*p* < 0.05) in the strawberry treatment group were observed for 3-(4-hydroxyphenyl)propanoic acid-3-sulfate, 3-hydroxyphenyl-gamma-valerolactones-4-sulfate, and hydroxycinnamic acid-O-glucuronide (^1^lacking reference standard; isomeric configuration unknown). Similar to the blueberry intervention, the absolute concentrations were relatively low. The largest change in concentration from baseline was for 3-hydroxyphenyl-gamma-valerolactones-4-sulfate (mean increase of ~45 nM; *p* = 0.002). The only low abundance metabolite that significantly changed in both interventions was 4-hydroxy-3,5-dimethoxyphenylacetic acid. 

Unconjugated hydroxybenzoic acids were identified with mean concentrations up to 5 µM, while 2-hydroxybenzoic acid, 3,4-dimethoxybenzoic acid, and 4-hydroxy-3-methoxybenzoic acid occurred in the highest abundance, although in some instances, these were only found following one of the interventions (blueberry or strawberry). Glucuronide conjugates appeared in the lowest abundance with mean concentrations generally below 250 nM. The most abundant glucuronide conjugates were identified as 4-hydroxybenzoic acid 3-*O*-glucuronide and 4-methoxybenzoic acid-3-*O*-glucuronide, at mean concentrations below 300 nM. Despite their modest concentrations, most metabolites did not show significant treatment effects.

## 4. Discussion

One of the major limitations in metabolomics is the substantial number of uncharacterized spectral features captured within a given scan, many of which are suspected to be either erroneous reaction artifacts or unknown biological conjugates [18]. Many of these uncharacterized spectral features are likely attributable to diet, as the contribution of plant-derived phytochemicals to the human metabolome is poorly characterized. The aim of the present work was to highlight the diversity of spectral features arising from diet, and particularly the contribution of (poly)phenols to the background metabolic signature. Berries are an effective model food to explore this, as they have significant phytochemical abundance and diversity and their consumption is linked to improved health. However, establishing the contribution of berry metabolites to the human metabolome is challenging, particularly as berries have a diverse phytochemical profile, which can be highly varied across varieties and cultivars. In the case of the blueberry and strawberry, the most consumed berries in the USA [7,8,9], similarities and differences were observed, and will be the basis for understanding their unique health benefits. Among the similarities is the clear restructuring of the “berry” metabolome (post-ingestion) as a result of human and microbial metabolism, wherein the metabolite profile in the body may become less diverse as various glycosides are cleaved from precursor structures and microbial metabolites are funneled to common products, such as phenolic acids. As there are no metabolomics methodologies or databases that fully capture the contribution of dietary phytochemicals to the human metabolome, the broad-spectrum metabolomics workflow described herein provides a potential first step forward in characterizing the contribution of plant phytochemicals to the human metabolome. It is important to note that the present manuscript is not primary research, but involves the development of a workflow and secondary analysis of clinical samples (NCT02035592 [4] and NCT02612090, unpublished) for exploring the contribution of phytochemicals to the human metabolome, which was not a focus of the initial berry intervention studies.

In the present metabolome analysis, there were more secondary plant metabolites reported in the literature for blueberries than for strawberries (Figure 2); however, this greater diversity may be the result of a greater number of publications, or differences in methodologies utilized among studies. Even though blueberries have a greater abundance (in number and content) of anthocyanins per se, strawberries are rich in ellagitannins and gallotannins, which are not present in blueberries, and strawberries have a greater diversity of procyanidins. Presently, the microbial catabolism of tannins/(poly)phenol polymers is uncharacterized and it is likely they contribute to the phenolic catabolite pools in ways not captured by the present literature. Clearly, the classes of phenolic metabolites found in the human metabolome are similar between berry interventions; however, the structures within each subclass, which change significantly, often differed. This is likely to reflect differences in hydroxy and methoxy functional groups of the precursor (poly)phenols within the berries, or differences in the microbiome of the individuals in the populations tested. Finally, as every study uses different methodologies, the findings reported in the literature are not definitive. More research is required in this area—particularly studies using the same methodologies and feeding different berries to the same study populations.

**Figure 7:**
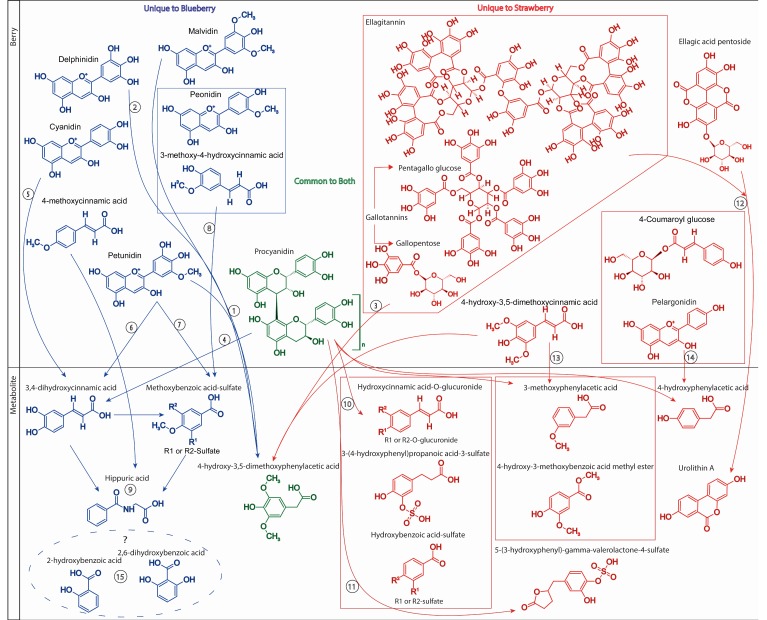
Proposed catabolism of precursor compounds to yield metabolites observed in the blueberry and strawberry interventions. The pathways of metabolites are speculative, and based on the structure and reported metabolism for monomeric (poly)phenols, as the microbial catabolic pathways for gallotannins, ellagitannins, and procyanidins are presently not fully characterized in humans. Isotope-labelling studies are required to characterize these pathways. Predictions were made using data from human studies feeding (poly)phenol-rich foods [5,10,16,24,25,31,36,37] and literature from environmental microbiology [5,34,35,38,39,40,41].

As suggested above, similar (poly)phenol subclasses are found in both blueberries and strawberries, for example, both contain anthocyanins and cinnamic acids in relatively high abundance. However, the hydroxylation and methylation patterns within these subclasses of (poly)phenols often differ. Strawberries contain primarily pelargonidin-type anthocyanins while blueberries contain a diversity of cyanidin, delphinidin, malvidin, peonidin, and petunidin *O*-glycosides (Figure 7). Further differences are observed in the subclasses of flavonols, flavones, flavan-3-ols, and hydroxybenzoic acids in blueberries and strawberries; however, these subclasses are reported as minor components, often providing less than 10 mg/100 g, cumulatively [7]. Perhaps the largest differences between these berries are in their abundance of reported (poly)phenol polymers. Even though blueberries and strawberries appear to have similar amounts (abundance) of procyanidins (between 60 and 130 mg/100 g), their diversity is reported to be much greater in the strawberry, which contains a larger number of trimer to tetramer polymers. Further, ellagitannins and gallotannins are essentially unique to strawberries. The higher abundance of polymers may serve as more persistent probiotic substrate to the gut microbiome, as these structures are unlikely to be catabolized to absorbable compounds as rapidly as (poly)phenol monomers. 

In the data extracted from the present literature, we observed large numbers of synonyms and inconsistent structural nomenclature or abbreviations, which is likely to hinder effective assimilation of data in online databases/repositories. For example, more than seven nonconformities for an anthocyanin glycoside are commonly reported. Using the anthocyanidin, cyanidin, as a base structure, its glucose conjugate is reported as either cyanidin glucoside, cyanidin-*O*-glucoside, cyanidin-3-*O*-glucoside, cyanidin-3-*O*-β-glucoside, cyanidin-3-*O*-β glucopyranoside, cyanidin 3-*O*-glucoside, or cyanidin hexoside. Acetyl glycosides further exaggerate this issue where various nomenclatures are used, for example, cyanidin-3-*O*-(6ʹ-acetyl)-galactoside, cyanidin-3-*O*-(6-acetyl)-galactoside, cyanidin-3-*O*-acetylgalactoside, cyanidin-3-acetylgalactoside, cyanidin-*O*-acetylgalactoside, and cyanidin acetylgalactoside. In addition, authors also use “*p*”, “*m*”, and “*o*”, signifying 4-, 3-, or 2-positional substitution. Further, phenolic acids are often described using common names, such as protocatechuic acid or syringic acid, which often have other reported synonyms in online databases. The use of structural and or IUPAC names would be less confusing for phenolic metabolites. For example, 3,4-dihydroxybenzoic acid and 3,5-dimethoxy-4-hydroxybenzoic acid should be used in place of protocatechuic acid and syringic acid, respectively. In this case, there are 10–15 synonyms reported for each of these simple phenolic structures in the Human Metabolome Database (HMDB. Retrieved September 16, 2019, from https://hmdb.ca/) [15] and PubChem (PubChem. Retrieved September 16, 2019, from https://pubchem.ncbi.nlm.nih.gov/) [19]. Finally, biological phase II conjugates are often named according to the synthetic or metabolic conjugation reaction with the precursor structure, as opposed to the structural formula of the reaction product. For example, 3,4-dihydroxybenzoic acid (or protocatechuic acid) can be conjugated with a glucuronic acid group as a result of phase II metabolism or synthetic reaction. This is often referred to as 3,4-dihydroxybenzoic acid-3-*O*-glucuronide (or protocatechuic acid-3-*O*-glucuronide) in online databases or synthetic reference standard manufacturers websites. However, the accurate structural name is 4-hydroxybenzoic acid-3-*O*-glucuronide. 3,4-dihydroxybenzoic acid (or protocatechuic acid) becomes a hydroxybenzoic acid-*O*-glucuronide once its 3-hydroxyl group is conjugated; therefore, it should no longer be referred to as a dihydroxybenozic acid-*O*-glucuronide (or protocatechuic acid-*O*-glucuronide). 

Following berry interventions, metabolites reached substantial concentrations in some participants (1–40 µM), with a diverse mixture of glucuronide and sulfate isomers ranging between 10 and 350 nM. Metabolites found in the highest abundance displayed the highest variability, which is likely to reflect differences in microbiome diversity, study design, and host intestinal transit. If structurally similar metabolites were to have additive or synergistic activities, there would be considerable potential to alter host physiology as their cumulative blood levels were observed at concentrations greater than 100 µM. The many instances of within-group effects, where metabolites were observed to increase from baseline in the absence of treatment or intervention effects (i.e., differences from control groups), suggest that high inter-individual variability precluded significant treatment effects. Changes in the metabolome resulting from other foods consumed during the intervention could also play a role.

The human metabolomes reported in the plasma/serum from the blueberry and strawberry intervention studies were similar in their diversity of classes of microbial metabolite; for example, benzoic, phenylacetic, 3-(phenyl)propanoic, and cinnamic acids. This is not surprising considering the diversity of microbial enzymes and pathways for aromatic compounds [20,21,22,23]. Individual metabolite structures were identified at concentrations as high as 10–25 µM for a few compounds, namely, 3-phenylpropanoic acid, 2-hydroxybenzoic acid, and hippuric acid, but more consistently observed between 50 and 250 nM (Figure 6) and with high variability. A number of metabolites were also found in low abundance, often below 25 nM, but were observed to significantly change from baseline as a result of the berry interventions (i.e., having significant treatment/intervention effects). However, despite reaching statistical significance, it is difficult to ascertain the biological relevance of these low abundance metabolites as single plasma/serum components, although, if their activities were additive or synergistic, their cumulative concentrations may reach a threshold level to deliver bioactivity. Cumulative activity has been reported using metabolites in in vitro cell culture models of vascular and anti-inflammatory activity [10,11,12,13,14]. Interestingly, 60% of the metabolites reported below 50 nM were sulfate or glucuronide conjugates, primarily of hydroxy benzoic, phenylacetic, cinnamic, and 3-(phenyl)propanoic acids; some glucuronide and sulfate conjugates of anthocyanins, catechins, and quercetin were also observed. Other studies feeding anthocyanin-rich supplements or products also identify significant increases in some of these common metabolites reported in the present study, at concentrations between 0.05 and 1 µM, including hippuric acid, 4-hydroxy-3-methoxybenzoic acid, 3-methoxybenzoic acid-4-*O*-glucuronide, and 3-hydroxyhippuric acid [4,10,16,24,25]. High levels of unconjugated phenolics were also observed in the present study and may be the result of compounds escaping phase II metabolism, possibly as a result of pathway saturation. Although drug polyphenol interactions have previously been reported [26], pathway saturation would likely have considerable metabolic consequences, and has not been reported previously in high berry consumers. More likely, the presence of unconjugated microbial metabolites indicates they are not major targets for metabolic detoxification or are possibly deconjugated in tissues post absorption. 

Of the metabolites found in the highest concentrations, only hippuric and 2-hydroxybenzoic acid were found to significantly increase relative to the placebo group following blueberry consumption [4], possibly as a result of the difference in the dose of anthocyanins within the berries fed across the two study interventions. The maximum dose of blueberry was 26 g freeze-dried material, equivalent to 150 g wet weight and providing 364 mg anthocyanins (879 mg total (poly)phenolics), while the strawberry intervention provided 50 g freeze-dried material, equivalent to approximately 500 g wet weight, providing 142 mg anthocyanins (451 mg total (poly)phenolics). Ultimately, the blueberry intervention provided a 60% higher abundance of anthocyanins and 49% more total polyphenols. Differences between the interventions were also likely a result of differing study design and metabolic differences between the study populations. Though the strawberry intervention delivered a lower dose of total (poly)phenols, metabolites were observed to shift significantly from baseline. However, in both interventions, many changes observed (i.e., increases from baseline) within the treatment groups followed a similar trend to that of the control groups, suggesting that other foods consumed during the intervention, or during the exclusion or washout periods, could influence metabolome fluctuation, which may obscure treatment effects.

Hydroxylation and methoxylation patterns of compounds observed within the classes of phenolic metabolites differed following blueberry and strawberry consumption, which likely reflects differences in precursor structures within the berries (Figure 7). Despite these differences, berry consumption in general leads to increased pools of small molecule metabolites already present at baseline, rather than the appearance of unique berry-derived metabolites. This indicates that common small molecule phenolic metabolites are likely derived from many plant/food sources, which cannot be “washed-out”, but rather fluctuate relative to dietary habits. This provides support to recent theories of (poly)phenols acting as hermetic response regulators [27], where low level exposure to microbial small molecule metabolites may trigger detoxification pathway enzymes or defense proteins at some threshold concentrations [28,29].

4-Hydroxy-3,5-dimethoxyphenylacetic acid was found to be significantly altered in both blueberry and strawberry interventions (Figure 7). This metabolite likely originated from different precursor structures with the blueberry and strawberry. 4-hydroxy-3,5-dimethoxyphenylacetic is a probable microbial catabolite of (1) malvidin- or petunidin-O-glycosides, as found in the blueberry [5,25,30]. Alternatively, following intestinal methylation, (2) delphinidin-*O*-glycosides could also be a contributing source. However, 4-hydroxy-3,5-dimethoxyphenylacetic is likely to be derived from the microbial catabolism of (3) ellagitannins, gallotannins, or (13) 4-hydroxy-3,5-dimethoxycinnamic acid following strawberry consumption [31,32,33,34,35]. The significant increase in 3,4-dihydroxycinnamic acid following blueberry intervention was also observed and likely derived from the microbial catabolism of (4) procyanidins [31] or (5) cyanidin-*O*-glycosides, or following de-methylation of (6) petunidin. The increase in methoxybenzoic acid sulfate was likely the consequence of microbial catabolism of (7) petunidin-*O*-glycosides or (8) 3-methoxy-4-hydroxycinnamic acid from the blueberry, while (9) hippuric acid is likely the terminal metabolite of multiple microbial catabolites [1,5].

Strawberry intervention resulted in the appearance of a number of human–microbial hybrid metabolites, including 3-(4-hydroxyphenyl)propanoic acid-3-sulfate, hydroxycinnamic acid-*O*-glucuronide, and hydroxybenzoic acid sulfate, which were likely derived from the metabolism of (poly)phenol polymers found in strawberries, such as (10) procyanidins (Figure 7) [31,32,33,34,35]. The metabolites that increased significantly following the strawberry intervention, which were not detected in the blueberry intervention samples, were 5-(3-hydroxyphenyl)-gamma-valerolactone-4-sulfate and urolithin A, known microbial metabolites of catechins and (11) procyanidins and (12) ellagic acid derivatives, respectively [5,34,35]. Tannin breakdown has been established primarily in soil microbial, fungal, and plant cell culture, where tannases liberate gallic acid, which is ultimately converted to pyrogallol before forming TCA cycle intermediates [32]. These products were not found in abundance in either intervention. Gallotannins are also reported to be converted to ellagic acid in microbial cell culture [33]. Urolithins are generally identified as the primary gut metabolites of ellagic acid and ellagitannins following consumption of ellagic acid-rich foods [34], and urolithin A was reported to increase significantly in the strawberry intervention, although its concentration was relatively low. Procyanidins have been reported in culture models to be catabolized forming 3,4-dihydroxyphenylacetic acid, 3-hydroxyphenylacetic acid, 4-hydroxyphenylacetic acid, and 3-(3-hydroxyphenyl)propanoic acid, in addition to 5-(3,4-dihydroxyphenyl)-gamma-valerolactone [35]. In the present study, 3-hydroxyphenylacetic acid was found in abundance in both blueberry and strawberry intervention samples, but did not change significantly, while 4-hydroxyphenylacetic acid and 5-(3,4-dihydroxyphenyl)-gamma-valerolactone and a sulfate metabolite of 5-(3,4-dihydroxyphenyl)-gamma-valerolactone did change significantly. Biotransformation of cranberry proanthocyanidins to cinnamic acids has been reported in microbial cell culture [31] and 3,4-dihydroxycinnamic acid significantly increased following blueberry intervention, while its glucuronide conjugate was significantly elevated in the strawberry intervention samples.

3-Methoxyphenylacetic acid and 4-hydroxy-3-methoxybenzoic acid methyl ester likely were microbial catabolites of (13) 4-hydroxy-3,5-dimethoxycinnamic acid (Figure 7), while 4-hydroxyphenylacetic acid was most likely a catabolite of (14) pelargonidin-O-glycosides or 4-coumaroyl glucose present in strawberries. Flavonoid degradation to phenylacetic acid has been reported in anaerobic microbial cell culture [42] and conversion of anthocyanins to phenylacetic, phenylpropanoic, cinnamic, and benzoic acids was reported previously [30] and was observed following both blueberry and strawberry interventions. Dihydroxycinnamic acid and cinnamic acid have been reported to be metabolized to (phenyl)propanoic acid in rats [38], and ultimately are converted to phenylacetic acid [43] and benzoic acids in microbial cell culture [39,40,41]. Further, oxidation of cinnamic acid to (hydroxyphenyl)propanoic acid has also been reported [43]. Cinnamic, (phenyl)propanoic, phenylacetic, and benzoic acids were all reported to increase significantly following blueberry and strawberry intervention, and differed in their hydroxylation and methoxylation patterns. It is unclear what the origins of (15) 2-hydroxybenzoic acid and 2,6-dihydroxybenzoic acid were in the blueberry intervention. Isotopic labelling studies would be required to confirm the origin of all these metabolites relative to their precursor phytochemicals within the berries.

Precursor polyphenols found in berries, such as anthocyanins or other coexistent flavonoids, do not appear to be the major circulating metabolites identified in fasting plasma/serum following berry consumption. The majority of metabolites appear to be derived from flavonoids and hydroxycinnamic acids (and possibly (poly)phenol polymers) degraded in the intestinal tract and/or converted by the microbiome to smaller phenolic structures, which persist in appreciable concentrations in human blood/plasma/serum. Significant changes in the metabolome following berry intervention often reflect small shifts in the metabolite pools at concentrations below 100 nM; the physiological relevance of this change is unclear. Adding berries to the diet clearly increases levels of hippuric, phenylacetic, 3-(phenyl)propanoic, and benzoic acids; however, biosignatures of metabolites reflecting berry consumption have yet to be verified. On the basis of limited data reported [4,10,16,24,25,36], it is unclear how berry consumption globally alters the human metabolome. A substantially greater number of metabolites are reported to change significantly when urine is used as a biological matrix, particularly with 24 h recovery, which may reflect a much more stable and concentrated metabolite pool than blood [4,36]. In the 24 h total urine pool from the blueberry intervention [4], large numbers of metabolites were found to change significantly between the treatment and control groups, including benzoylglutamic acid, chlorogenic acid, hippuric acid, trans-3-hydroxycinnamic acid, 2-hydroxybenzoic acid, 3-hydroxybenzoic acid, 3-hydroxyhippuric acid, 3-hydroxyphenylpropionic acid, 4-hydroxyhippuric acid, 3,4-dihydroxycinnamic acid, 3,4-dihydroxyphenylacetic acid, 3-(3,4-dihydroxyphenyl)propionic acid, 3-hydroxybenzoic acid-4-sulfate, 4-hydroxybenzoic acid-3-sulfate, 4-methoxybenzoic acid-3-sulfate, 3-hydroxy-4-methoxycinnamic acid, 2,5-dihydroxybenzoic acid, 3,5-dimethoxybenzoic acid methyl ester, 4-methoxybenzoic acid-3-glucuronide, 4-hydroxy-3-methoxybenzoic acid, 4-hydroxy-3-methoxycinnamic acid, 4-hydroxy-3-methoxyphenylacetic acid, 3-(4-hydroxy-3-methoxyphenyl)propionic acid, 4-hydroxy-3,5-dimethoxybenzoic acid, 4-hydroxy-3,5-dimethoxycinnamic acid, and 3,4,5-trimethoxybenzaldehyde. Meanwhile, only four metabolites significantly increased in the blood. Although the strawberry intervention analyzed in the present work did not collect 24 h urine pools, similar observations have been reported in the literature [1]. For example, a recent raspberry intervention, which has a similar anthocyanin profile to blueberries and strawberries, reported urine and breast milk to show greater abundance of metabolites than blood [37]. Regardless of an often lack of statistical significance (treatment effect), the metabolites reported in the present study reflect those found in highest abundance in the metabolomes of the samples from the feeding trials (Figure 6). These provide context when interpreting flux in the metabolome post consumption. This is important as most work in this field focuses on what changes significantly, and even though this present work comments on what changes significantly, more importantly, it highlights the extreme levels of some proposed berry metabolites that are already present in abundance in baseline samples. This suggests that small, but significant changes of any metabolite resulting from an intervention may have limited relevance, relative to the substantial abundance of these metabolites already present in the background metabolome. Future studies should, therefore, focus either on large nanomolar changes in concentration (e.g., >100) or overall flux in the metabolome.

Owing to the similarities in dietary precursor phytochemicals, changes in short-term dietary patterns are unlikely to shift phenolic catabolite profiles appreciably without significant dietary control or fluctuation in the microbiome. Acute change in metabolome could simply reflect altered clearance kinetics of microbial metabolites resulting from differences in gastric emptying and intestinal transit. Total 24 h urine sampling may provide more consistent metabolome data when the acute response is being investigated or in less controlled cohort studies. Despite these differences in urine and plasma/serum metabolites, the diversity and abundance of dietary (poly)phenol metabolites highlight the value of characterizing their contribution to the human metabolome, particularly as some microbial metabolites are identical to endogenous metabolites and pharmacoactive agents. As there are many food sources rich in (poly)phenols, such as berries, coffee, tea, wine, potato, nuts, apples, and plums; characterizing the contribution of (poly)phenols and their microbial metabolites to the human metabolome is complex, but important, as they are likely to influence metabolomic assessment. Such datasets are critical to more accurately and specifically determine their potential to improve health.

## 5. Conclusions

The impact of berry phytochemicals on the human metabolome is challenging, requiring broad-spectrum metabolomics and data from multiple studies of consistent design. As berry consumption appears to cause fluctuation in preexisting pools of small molecule metabolites, rather than the appearance of unique berry derived metabolites, it may be more important to map metabolome flux rather than to trace any single metabolite, particularly when exploring bioactivity.

## Figures and Tables

**Table 1:** Literature review search strategy.

Categories	Search Terms	PubMed	WebofSci
		Hits	Hits
1. Berry	Strawberry, blueberry, Fragaria, Vaccinium	6614	18,534
2. Phenolics	Phenols, Flavonoids, Flavonoid *, Polyphenols	336,958	269,334
3. ADME	Metabolism, Pharmacokinetics	2,129,074	1,998,115
4. Analytical technique/type	Chromatography, Mass spectrometry	586,140	1,142,675
5. Controlled/clinical intervention	Clinical Trial, Fluids and secretions, Clinical laboratory techniques	3,624,193	2,364,049
6. Food AND phenolics AND ADME AND (analytical technique/type OR controlled/clinical intervention)	#1 AND #2 AND #3 AND (#4 OR #5)	98	271

Search strategy utilized standard Boolean operators in order to combine search terms or filter results. OR allows hits with either/any search terms to be included. AND requires both/all search terms to be included. * represents a “wildcard” character used to indicate root word truncation. HITS denotes the number of unique records returned. ADME, absorption, distribution, metabolism, elimination.

**Table 2:** Methodology history of use.

Intervention	Biospecimen	Number Samples	Number Reference Standards	Putative	Transitions	Number Metabolites Identified
^1^ Strawberries	Plasma	146	98	44	531	142
^2^ Blueberries	Serum	544	102	74	557	176
Total		690				

^1^ Strawberry intervention feeding equivalent of 3.5 cups of strawberries (50 g freeze-dried powder) for four weeks (unpublished); ^2^ blueberry intervention feeding equivalent of ½ or 1 cups of blueberries (75 g and 150 g freeze-dried powder, respectively) for six months [4].

## Supplementary Materials

The following are available online at https://www.mdpi.com/1420-3049/24/23/4220/s1, Figure S1. Inclusion and exclusion criteria: In-out criteria flow diagram; Table S2. Methodology reference standard and MS/MS transition list; Text S3. Excluded references at title evaluation stage; Text S4. Excluded references at abstract evaluation stage; Text S5. Excluded references at full text evaluation stage; Text S6. Included manuscript reference list.

## Abbreviations

Advanced scheduled MRM (ADsMRM), coefficient of variation (CV), limit of detection (LOD), lower limit of quantification (LLOQ), middle quality control (MCQ), multiple reaction monitoring (MRM), multi-stage tandem mass spectrometry (MS^n^), scheduled MRM (sMRM), solid phase extraction (SPE), tandem mass spectrometry (MS/MS), ultra-high performance liquid chromatography-tandem mass spectrometry (UHPLC-MS/MS), upper limit of quantitation (ULOQ).

## References

[1] Kay C.D., Pereira-Caro G., Ludwig I.A., Clifford M.N., Crozier A. (2017). Anthocyanins and flavanones are more bioavailable than previously perceived: A review of recent evidence. Annu. Rev. Food Sci. Technol..

[2] Tsuda T. (2011). Dietary anthocyanin-rich plants: Biochemical basis and recent progress in health benefits studies. Mol. Nutr. Food Res..

[3] Yang B., Kortesniemi M. (2015). Clinical evidence on potential health benefits of berries. Curr. Opin. Food Sci..

[4] Curtis P.J., van der Velpen V., Berends L., Jennings A., Feelisch M., Umpleby A.M., Evans M., Fernandez B.O., Meiss M.S., Minnion M. (2019). Blueberries improve biomarkers of cardiometabolic function in participants with metabolic syndrome—results from a 6-month, double-blind, randomized controlled trial. Am. J. Clin. Nutr..

[5] Williamson G., Kay C.D., Crozier A. (2018). The Bioavailability, Transport, and Bioactivity of Dietary Flavonoids: A Review from a Historical Perspective. Compr. Rev. Food Sci. Food Saf..

[6] Zhong S., Sandhu A., Edirisinghe I., Burton-Freeman B. (2017). Characterization of Wild Blueberry Polyphenols Bioavailability and Kinetic Profile in Plasma over 24-h Period in Human Subjects. Mol. Nutr. Food Res..

[7] USDA (2011). USDA Database for the Flavonoid Content of Selected Foods (Release 3.0).

[8] Bowman S., Clemens J.C., Friday J.E., Lynch K.L., LaComb R.P., Moshfegh A.J. (2017). Food Patterns Equivalents Intakes by Americans: What We Eat in America, NHANES 2003-04 and 2013-14.

[9] Neveu V., Perez-Jiménez J., Vos F., Crespy V., du Chaffaut L., Mennen L., Knox C., Eisner R., Cruz J., Wishart D. (2010). Phenol-Explorer: An online comprehensive database on polyphenol contents in foods. Database.

[10] de Ferrars R.M., Cassidy A., Curtis P., Kay C.D. (2014). Phenolic metabolites of anthocyanins following a dietary intervention study in post-menopausal women. Mol. Nutr. Food Res..

[11] Warner E.F., Zhang Q., Raheem K.S., O’Hagan D., O’Connell M.A., Kay C.D. (2016). Common phenolic metabolites of flavonoids, but not their unmetabolized precursors, reduce the secretion of vascular cellular adhesion molecules by human endothelial cells. J. Nutr..

[12] Warner E.F., Smith M.J., Zhang Q., Raheem K.S., O’Hagan D., O’Connell M.A., Kay C.D. (2017). Signatures of anthocyanin metabolites identified in humans inhibit biomarkers of vascular inflammation in human endothelial cells. Mol. Nutr. Food Res..

[13] Warner E., Rodriguez-Ramiro I., O’Connell M., Kay C. (2018). Cardiovascular Mechanisms of Action of Anthocyanins May Be Associated with the Impact of Microbial Metabolites on Heme Oxygenase-1 in Vascular Smooth Muscle Cells. Molecules.

[14] di Gesso J.L., Kerr J.S., Zhang Q., Raheem S., Yalamanchili S.K., O’hagan D., Kay C.D., O’connell M.A. (2015). Flavonoid metabolites reduce tumor necrosis factor-α secretion to a greater extent than their precursor compounds in human THP-1 monocytes. Mol. Nutr. Food Res..

[15] Wishart D.S., Tzur D., Knox C., Eisner R., Guo A.C., Young N., Cheng D., Jewell K., Arndt D., Sawhney S. (2007). HMDB: The Human Metabolome Database. Nucleic Acids Res..

[16] Nieman D., Kay C., Rathore A., Grace M., Strauch R., Stephan E., Sakaguchi C., Lila M. (2018). Increased Plasma Levels of Gut-Derived Phenolics Linked to Walking and Running Following Two Weeks of Flavonoid Supplementation. Nutrients.

[17] Nieman D.C., Ramamoorthy S., Kay C.D., Goodman C.L., Capps C.R., Shue Z.L., Heyl N., Grace M.H., Lila M.A. (2017). Influence of Ingesting a Flavonoid-Rich Supplement on the Metabolome and Concentration of Urine Phenolics in Overweight/Obese Women. J. Proteome Res..

[18] Baran R., Northen T.R. (2013). Robust automated mass spectra interpretation and chemical formula calculation using mixed integer linear programming. Anal. Chem..

[19] Kim S., Thiessen P.A., Bolton E.E., Bryant S.H. (2015). PUG-SOAP and PUG-REST: Web services for programmatic access to chemical information in PubChem. Nucleic Acids Res..

[20] Kamimura N., Takahashi K., Mori K., Araki T., Fujita M., Higuchi Y., Masai E. (2017). Bacterial catabolism of lignin-derived aromatics: New findings in a recent decade: Update on bacterial lignin catabolism. Environ. Microbiol. Rep..

[21] Krastanov A., Alexieva Z., Yemendzhiev H. (2013). Microbial degradation of phenol and phenolic derivatives. Eng. Life Sci..

[22] Mishra V.K., Kumar N. (2017). Microbial Degradation of Phenol: A Review. J. Water Pollut. Purif. Res..

[23] Jimenez N., Reveron I., Esteban-Torres M., Lopez de Felipe F., de Las Rivas B., Munoz R. (2014). Genetic and biochemical approaches towards unravelling the degradation of gallotannins by Streptococcus gallolyticus. Microb. Cell Factories.

[24] Rodriguez-Mateos A., Del Pino-Garcia R., George T.W., Vidal-Diez A., Heiss C., Spencer J.P. (2014). Impact of processing on the bioavailability and vascular effects of blueberry (poly)phenols. Mol. Nutr. Food Res..

[25] Rodriguez-Mateos A., Rendeiro C., Bergillos-Meca T., Tabatabaee S., George T.W., Heiss C., Spencer J.P. (2013). Intake and time dependence of blueberry flavonoid-induced improvements in vascular function: A randomized, controlled, double-blind, crossover intervention study with mechanistic insights into biological activity. Am. J. Clin. Nutr..

[26] Joshua D.L., Shengmin S., Anthony Y.H.L., Chung S.Y. (2007). Metabolism of Dietary Polyphenols and Possible Interactions with Drugs. Curr. Drug Metab..

[27] Franco R., Navarro G., Martinez-Pinilla E. (2019). Hormetic and Mitochondria-Related Mechanisms of Antioxidant Action of Phytochemicals. Antioxidants.

[28] Calabrese V., Cornelius C., Dinkova-Kostova A.T., Calabrese E.J. (2009). Vitagenes, cellular stress response, and acetylcarnitine: Relevance to hormesis. Biofactors.

[29] Gallardo-Fernandez M., Hornedo-Ortega R., Cerezo A.B., Troncoso A.M., Garcia-Parrilla M.C. (2019). Melatonin, protocatechuic acid and hydroxytyrosol effects on vitagenes system against alpha-synuclein toxicity. Food Chem. Toxicol..

[30] de Ferrars R.M., Czank C., Zhang Q., Botting N.P., Kroon P.A., Cassidy A., Kay C.D. (2014). The pharmacokinetics of anthocyanins and their metabolites in humans. Br. J. Pharmacol..

[31] Rupasinghe H.P.V., Parmar I., Neir S.V. (2019). Biotransformation of Cranberry Proanthocyanidins to Probiotic Metabolites by Lactobacillus rhamnosus Enhances Their Anticancer Activity in HepG2 Cells In Vitro. Oxid Med. Cell Longev..

[32] Li M., Kai Y., Qiang H., Dongying J. (2006). Biodegradation of gallotannins and ellagitannins. J. Basic Microbiol..

[33] Gauri S.S., Mandal S.M., Atta S., Dey S., Pati B.R. (2013). Novel route of tannic acid biotransformation and their effect on major biopolymer synthesis in Azotobacter sp. SSB81. J. Appl. Microbiol..

[34] Tomas-Barberan F.A., Garcia-Villalba R., Gonzalez-Sarrias A., Selma M.V., Espin J.C. (2014). Ellagic acid metabolism by human gut microbiota: Consistent observation of three urolithin phenotypes in intervention trials, independent of food source, age, and health status. J. Agric. Food Chem..

[35] Appeldoorn M.M., Vincken J.P., Aura A.M., Hollman P.C., Gruppen H. (2009). Procyanidin dimers are metabolized by human microbiota with 2-(3,4-dihydroxyphenyl)acetic acid and 5-(3,4-dihydroxyphenyl)-gamma-valerolactone as the major metabolites. J. Agric. Food Chem..

[36] Sandhu A.K., Miller M.G., Thangthaeng N., Scott T.M., Shukitt-Hale B., Edirisinghe I., Burton-Freeman B. (2018). Metabolic fate of strawberry polyphenols after chronic intake in healthy older adults. Food Funct..

[37] Zhang X., Sandhu A., Edirisinghe I., Burton-Freeman B. (2018). An exploratory study of red raspberry (Rubus idaeus L.) (poly) phenols/metabolites in human biological samples. Food Funct..

[38] Gonthier M.P., Verny M.A., Besson C., Remesy C., Scalbert A. (2003). Chlorogenic acid bioavailability largely depends on its metabolism by the gut microflora in rats. J. Nutr..

[39] Campillo T., Renoud S., Kerzaon I., Vial L., Baude J., Gaillard V., Bellvert F., Chamignon C., Comte G., Nesme X. (2014). Analysis of hydroxycinnamic acid degradation in Agrobacterium fabrum reveals a coenzyme A-dependent, beta-oxidative deacetylation pathway. Appl. Environ. Microbiol..

[40] Gasson M.J., Kitamura Y., McLauchlan W.R., Narbad A., Parr A.J., Parsons E.L., Payne J., Rhodes M.J., Walton N.J. (1998). Metabolism of ferulic acid to vanillin. A bacterial gene of the enoyl-SCoA hydratase/isomerase superfamily encodes an enzyme for the hydration and cleavage of a hydroxycinnamic acid SCoA thioester. J. Biol. Chem..

[41] Karmakar B., Vohra R.M., Nandanwar H., Sharma P., Gupta K.G., Sobti R.C. (2000). Rapid degradation of ferulic acid via 4-vinylguaiacol and vanillin by a newly isolated strain of bacillus coagulans. J. Biotechnol..

[42] Schoefer L., Mohan R., Schwiertz A., Braune A., Blaut M. (2003). Anaerobic degradation of flavonoids by Clostridium orbiscindens. Appl. Environ. Microbiol..

[43] Lapadatescu C., Ginies C., Le Quere J.L., Bonnarme P. (2000). Novel scheme for biosynthesis of aryl metabolites from L-phenylalanine in the fungus Bjerkandera adusta. Appl. Environ. Microbiol..

