# Supplementary material for: Contribution of Berry Polyphenols to the Human Metabolome
Source: Molecules. 2019 Nov 20;24(23):4220. doi: 10.3390/molecules24234220 (PMC6930569; doi:10.3390/molecules24234220)
Supplement: Supplementary file 1 [file molecules-24-04220-s001.zip › Supplement 2 Table1.docx]

**Table S1.** Reference Standard list and number of qualitative MS transitions utilized

| **Full Name** | **CAS** | **Reference Standard** | **Relative Quantifier** | **Transitions** |
| --- | --- | --- | --- | --- |
| (-)-quinic acid | 201-072-8 | ✓ | reference standard | **2** |
| 2-methylhippuric acid | 42013-20-7 | ✓ | reference standard | 2 |
| 3-(2,4-dihydroxyphenyl)propanoic acid | 5631-68-5 | ✓ | reference standard | 4 |
| 2,4,6-trihydroxybenzaldehyde | 487-70-7 | ✓ | reference standard | 5 |
| 2,3-dihydroxybenzoic acid | 303-38-8 | ✓ | reference standard | 4 |
| 4-methoxycinnamic acid-3-o-glucuronide | 1065272-10-7 | ✓ | reference standard | 6 |
| 3,4-dihydroxyphenylacetic acid | 102-32-9 | ✓ | reference standard | 3 |
| urolithin A | 1143-70-0 | ✓ | reference standard | 5 |
| urolithin B | 1139-83-9 | ✓ | reference standard | 3 |
| 5-hydroxybenzoic acid-3-sulfate | synthetic | ✓ | reference standard | 2 |
| 3-(5-hydroxyphenyl)propanoic acid-3-o-glucuronide | synthetic | ✓ | reference standard | 3 |
| 3-(5-hydroxyphenyl)propanoic acid-3-sulfate | synthetic | ✓ | reference standard | 4 |
| cinnamic acid-4-o-glucuronide | 214689-30-2 | ✓ | reference standard | 2 |
| 3-hydroxycinnamic acid-4-o-glucuronide | 1093679-71-0 | ✓ | reference standard | 6 |
| 3-hydroxybenzoic acid-4-o-glucuronide | synthetic | ✓ | reference standard | 4 |
| 3-o-caffeoylquinic acid | 327-97-9 | ✓ | reference standard | 5 |
| 4-o-caffeoylquinic acid | 905-99-7 | ✓ | reference standard | 4 |
| 3-methoxyphenylpropanoic acid-4-sulfate | 86321-33-7 | ✓ | reference standard | 5 |
| 4-methoxycinnamic acid-3-sulfate | synthetic | ✓ | reference standard | 3 |
| 3-(4-hydroxyphenyl)propionic acid-3-sulfate | 1187945-70-5 | ✓ | reference standard | 4 |
| 4-methoxyphenylpropanoic acid-3-o-glucuronide | 1187945-72-7 | ✓ | reference standard | 6 |
| 3,4-hydroxybenzoic acid-3-o-sulfate | synthetic | ✓ | reference standard | 4 |
| 1,3-benzenediol | 108-46-3 | ✓ | reference standard | 3 |
| trans cinnamic acid | 140-10-3 | ✓ | reference standard | 1 |
| 3-(3-methoxyphenyl)propionic acid | 10516-71-9 | ✓ | reference standard | 4 |
| alpha hydroxyhippuric acid | 16555-77-4 | ✓ | reference standard | 4 |
| 3,5-dimethoxybenzoic acid | 1132-21-4 | ✓ | reference standard | 3 |
| 4-methylhippuric acid | 27115-50-0 | ✓ | reference standard | 5 |
| 3-(4-hydroxy-3-methoxyphenyl)propionic acid | 1135-23-5 | ✓ | reference standard | 5 |
| 2,3,4-trimethoxyphenylacetic acid | 22480-91-7 | ✓ | reference standard | 4 |
| 3,4-dihydroxybenzoic acid methyl ester | 2150-43-8 | ✓ | reference standard | 4 |
| 3,5-dihydroxybenzoic acid methyl ester | 2150-44-9 | ✓ | reference standard | 4 |
| 3-hydroxybenzoic acid methyl ester | 19438-10-9 | ✓ | reference standard | 3 |
| 4-hydroxy-3-methoxybenzoic acid methyl ester | 3943-74-6 | ✓ | reference standard | 4 |
| 4-hydroxybenzoic acid methyl ester | 99-76-3 | ✓ | reference standard | 3 |
| 2,4-dihydroxybenzaldehyde | 95-01-2 | ✓ | reference standard | 4 |
| 3,4,5-trihydroxybenzaldehyde | 13677-79-7 | ✓ | reference standard | 3 |
| 3,4-dihydroxybenzaldehyde | 139-85-5 | ✓ | reference standard | 3 |
| 3,5-dihydroxybenzaldehyde | 26153-38-8 | ✓ | reference standard | 2 |
| 3-hydroxy-4-methoxybenzaldehyde | 621-59-0 | ✓ | reference standard | 3 |
| 3-hydroxybenzaldehyde | 100-83-4 | ✓ | reference standard | 3 |
| 4-hydroxybenzaldehyde | 123-08-0 | ✓ | reference standard | 3 |
| 2,4-dihydroxybenzoic acid | 89-86-1 | ✓ | reference standard | 3 |
| 2,5-dihydroxybenzoic acid | 490-79-9 | ✓ | reference standard | 3 |
| 2-hydroxy-4-methoxybenzoic acid | 2237-36-7 | ✓ | reference standard | 3 |
| 2-hydroxy-6-methoxybenzoic acid | 3147-64-6 | ✓ | reference standard | 2 |
| 2-hydroxybenzoic acid | 69-72-7 | ✓ | reference standard | 3 |
| 3,4,5-trihydroxybenzoic acid | 149-91-7 | ✓ | reference standard | 3 |
| 3,4-dihydroxybenzoic acid | 99-50-3 | ✓ | reference standard | 3 |
| 3-hydroxy-4-methoxybenzoic acid | 645-08-9 | ✓ | reference standard | 3 |
| 3-hydroxybenzoic acid | 99-06-9 | ✓ | reference standard | 3 |
| 3-methoxybenzoic acid-4-o-glucuronide | synthetic | ✓ | reference standard | 3 |
| 3-methoxybenzoic acid-4-sulfate | synthetic | ✓ | reference standard | 3 |
| 4-hydroxy-3-methoxybenzoic acid | 121-34-6 | ✓ | reference standard | 5 |
| 4-hydroxybenzoic acid | 99-96-7 | ✓ | reference standard | 2 |
| 4-methoxybenzoic acid-3-o-glucuronide | synthetic | ✓ | reference standard | 3 |
| 4-methoxybenzoic acid-3-sulfate | synthetic | ✓ | reference standard | 3 |
| benzoic acid | 65-85-0 | ✓ | reference standard | 1 |
| 4-hydroxybenzoic acid-4-sulfate | synthetic | ✓ | reference standard | 2 |
| 2-hydroxycinnamic acid | 614-60-8 | ✓ | reference standard | 3 |
| 3,4-dihydroxycinnamic acid | 331-39-5 | ✓ | reference standard | 3 |
| 3-hydroxy-4-methoxycinnamic acid | 537-73-5 | ✓ | reference standard | 3 |
| 4-hydroxy-3-methoxycinnamic acid | 537-98-4 | ✓ | reference standard | 3 |
| 4-hydroxycinnamic acid | 7400-08-0 | ✓ | reference standard | 3 |
| trans 3-hydroxycinnamic acid | 14755-02-3 | ✓ | reference standard | 3 |
| 3-methylhippuric acid | 27115-49-7 | ✓ | reference standard | 3 |
| 4-hydroxyhippuric acid | 2482-25-9 | ✓ | reference standard | 3 |
| hippuric acid | 495-69-2 | ✓ | reference standard | 3 |
| 1,2-dihydroxybenzene | 120-80-9 | ✓ | reference standard | 3 |
| 4-hydroxy-3-methoxyphenylacetic acid | 306-08-1 | ✓ | reference standard | 1 |
| 3-(3,4-dihydroxyphenyl)propionic acid | 1078-61-1 | ✓ | reference standard | 3 |
| 3-(3-hydroxy-4-methoxyphenyl)propionic acid | 1135-15-5 | ✓ | reference standard | 3 |
| 3-(4-methoxyphenyl)propanoic acid-3-sulfate | synthetic | ✓ | reference standard | 3 |
| 3-(3-hydroxyphenyl)propionic acid | 621-54-5 | ✓ | reference standard | 2 |
| 3-(4-hydroxyphenyl)propionic acid | 501-97-3 | ✓ | reference standard | 3 |
| 3-phenylpropionic acid | 501-52-0 | ✓ | reference standard | 1 |
| rosmarinic acid | 20283-92-5 | ✓ | reference standard | 3 |
| resveratrol | 501-36-0 | ✓ | reference standard | 5 |
| 3-methoxyphenylacetic acid | 1798-09-0 | ✓ | reference standard | 2 |
| 3-hydroxyhippuric acid | 1637-75-8 | ✓ | reference standard | 4 |
| n-benzoylglutamic acid | 6094-36-6 | ✓ | reference standard | 4 |
| 3-hydroxy-4-methoxyphenylacetic acid | 1131-94-8 | ✓ | reference standard | 2 |
| 3-hydroxyphenylacetic acid | 621-37-4 | ✓ | reference standard | 3 |
| 3-methoxycinnamic acid | 6099-04-3 | ✓ | reference standard | 3 |
| 4-methoxycinnamic acid | 830-09-1 | ✓ | reference standard | 4 |
| 2-hydroxy-4-methoxybenzaldehyde | 673-22-3 | ✓ | reference standard | 3 |
| quercetin-3-o-glucuronide | 22688-79-5 | ✓ | reference standard | 7 |
| 3-methoxyphenylpropanoic acid-4-o-glucuronide | 86321-28-0 | ✓ | reference standard | 5 |
| 3-methoxyphenylacetic acid-4-sulfate | 38339-06-8 | ✓ | reference standard | 5 |
| 3-hydroxyphenyl-gamma-valerolactone-4-sulfate | synthetic | ✓ | reference standard | 4 |
| benzoic acid-diglucuronide | x | x | 3-methoxybenzoic acid-4-O-glucuronide | 4 |
| hydroxybenzyldehyde-o-glucuronide | x | x | 3-methoxybenzoic acid-4-O-glucuronide | 5 |
| hydroxyphenylacetic acid-o-glucuronide | x | x | 4-methoxybenzoic acid-3-O-glucuronide | 4 |
| 5-o-caffeoylquinic acid | x | x | 3-O-caffeoylquinic acid | 5 |
| catechin-o-glucuronide | x | x | quercetin-3-O-glucuronide | 5 |
| hydroxyphenylvalerolactone-sulfate | x | x | 3-hydroxyphenyl-gamma-valerolactone-4-sulfate | 4 |
| methoxycinnamic acid-sulfoglucuronide | x | x | 3-methoxybenzoic acid-4-sulfate | 5 |
| phenylvalerolactone-sulfoglucuronide | x | x | 3-hydroxyphenyl-gamma-valerolactone-4-sulfate | 5 |
| phenylvalerolactone-sulfate | x | x | 3-hydroxyphenyl-gamma-valerolactone-4-sulfate | 3 |
| hydroxyphenyl-gamma-valerolactone-o-glucuronide | x | x | 3-hydroxyphenyl-gamma-valerolactone-4-sulfate | 6 |
| 3-caffeoylquinic acid-o-glucuronide | x | x | 3-hydroxyphenyl-gamma-valerolactone-4-sulfate | 6 |
| epicatechin-o-glucuronide | x | x | quercetin-3-O-glucuronide | 6 |
| gallocatechin-o-glucuronide | x | x | quercetin-3-O-glucuronide | 6 |
| 3-o-feruloylquinic acid | x | x | 3-O-caffeoylquinic acid | 3 |
| 4-o-feruloylquinic acid | x | x | 3-O-caffeoylquinic acid | 3 |
| 5-o-feruloylquinic acid | x | x | 3-O-caffeoylquinic acid | 3 |
| hydroxy-methoxyphenylacetic acid-o-glucuronide | x | x | 3-methoxyphenylpropanoic acid-4-O-glucuronide | 5 |
| phenylpropanoic acid-o-glucuronide | x | x | 3-methoxyphenylpropanoic acid-4-O-glucuronide | 5 |
| dihydroxybenzoic acid-sulfate | x | x | 5-hydroxybenzoic acid-3-sulfate | 4 |
| trihydroxybenzaldehyde-sulfate | x | x | 5-hydroxybenzoic acid-3-sulfate | 4 |
| hippuric acid-sulfate | x | x | 5-hydroxybenzoic acid-3-sulfate | 4 |
| hydroxyhippuric acid | x | x | 4-hydroxyhippuric acid | 3 |
| dihydroxybenzoic acid | x | x | 2,4-dihydroxybenzoic acid | 3 |
| hydroxy-methoxybenzoic acid-sulfate | x | x | 5-hydroxybenzoic acid-3-sulfate | 4 |
| dihydroxyphenylacetic acid | x | x | 3-hydroxyphenylacetic acid | 4 |
| hydroxybenzoic acid-sulfate | x | x | 5-hydroxybenzoic acid-3-sulfate | 4 |
| urolithin A-O-glucuronide | x | x | urolithin A | 6 |
| urolithin A-O-glucuronide | x | x | urolithin A | 6 |
| hydroxycinnamic acid-o-glucuronide | x | x | 3-hydroxycinnamic acid-4-O-glucuronide | 6 |
| hydroxycinnamic acid-o-glucuronide | x | x | 3-hydroxycinnamic acid-4-O-glucuronide | 6 |
| 4-hydroxy-2-methoxybenzaldehyde | 18278-34-7 | ✓ | reference standard | 3 |
| 3,5-dihydroxybenzoic acid | 99-10-5 | ✓ | reference standard | 3 |
| 4-hydroxy-3,5-dimethoxybenzoic acid | 530-57-4 | ✓ | reference standard | 3 |
| hippuric acid methyl ester | 1205-08-9 | ✓ | reference standard | 5 |
| 1,2-dihydroxy-4-methylbenzene | 452-86-8 | ✓ | reference standard | 3 |
| 4-hydroxy-3,5-dimethoxyphenylacetic acid | 4385-56-2 | ✓ | reference standard | 5 |
| 4-hydroxyphenylacetic acid | 156-38-7 | ✓ | reference standard | 2 |
| 4-methoxyphenylacetic acid | 104-01-8 | ✓ | reference standard | 3 |
| methoxycinnamic acid-o-glucuronide | x | x | 4-methoxybenzoic acid-3-O-glucuronide | 6 |
| 3-(4-methoxyphenyl)propanoic acid-3-o-glucuronide | x | x | 4-methoxybenzoic acid-3-O-glucuronide | 4 |
| methoxyphenylacetic acid-o-glucuronide | x | x | 4-methoxybenzoic acid-3-O-glucuronide | 3 |
| hydroxy-methoxybenzyldehyde-o-glucuronide | x | x | 4-methoxybenzoic acid-3-O-glucuronide | 4 |
| ascorbic acid-sulfate | x | x | 3,4-dihydroxybenzoic acid | 6 |
| methoxycinnamic acid-sulfate | x | x | 5-hydroxybenzoic acid-3-sulfate | 4 |
| pyridoxic acid sulfate | x | x | 5-hydroxybenzoic acid-3-sulfate | 5 |
| dihydroxy-methoxycinnamic acid-sulfate | x | x | 5-hydroxybenzoic acid-3-sulfate | 6 |
| hippuric acid sulfate methyl ester | x | x | 5-hydroxybenzoic acid-3-sulfate | 6 |
| methoxybenzoic acid sulfate | x | x | 5-hydroxybenzoic acid-3-sulfate | 5 |
| methoxyphenylpropanoic acid-sulfate | x | x | 5-hydroxybenzoic acid-3-sulfate | 4 |
| hydroxy-methoxybenzaldehyde sulfate | x | x | 5-hydroxybenzoic acid-3-sulfate | 4 |
| valeric acid-sulfate | x | x | 5-hydroxybenzoic acid-3-sulfate | 5 |
| hydroxyphenylacetic acid-sulfate | x | x | 5-hydroxybenzoic acid-3-sulfate | 4 |
