# Supplementary material for: Contribution of Berry Polyphenols to the Human Metabolome
Source: Molecules. 2019 Nov 20;24(23):4220. doi: 10.3390/molecules24234220 (PMC6930569; doi:10.3390/molecules24234220)
Supplement: Supplementary file 1 [file molecules-24-04220-s001.zip › Supplement 3.docx]

**S3. Excluded references at title evaluation stage**

| 1. Abdulazeez, S. S. and P. Ponnusamy (2016). "Antioxidant and hypoglycemic activity of strawberry fruit extracts against alloxan induced diabetes in rats." Pakistan Journal of Pharmaceutical Sciences 29(1): 255-260. |
| --- |
| 1. Ahmed, M., et al. (2014). "The Protective Effects of a Polyphenol-Enriched Protein Powder on Exercise-Induced Susceptibility to Virus Infection." Phytotherapy Research 28(12): 1829-1836. |
| 1. Akhatou, I., et al. (2017). "Application of Targeted Metabolomics to Investigate Optimum Growing Conditions to Enhance Bioactive Content of Strawberry." J Agric Food Chem 65(43): 9559-9567. |
| 1. Al Hamimi, S., et al. (2017). "Alterations in the plasma metabolite profile associated with improved hepatic function and glycemia in mice fed lingonberry supplemented high-fat diets." Mol Nutr Food Res 61(3): 10. |
| 1. Aqil, F., et al. (2014). "Detection of Anthocyanins/Anthocyanidins in Animal Tissues." J Agric Food Chem 62(18): 3912-3918. |
| 1. Ayoub, M., et al. (2016). "Antioxidants and bioactivities of free, esterified and insoluble-bound phenolics from berry seed meals." Food Chem 197(Pt A): 221-232. |
| 1. Azorin-Ortuno, M., et al. (2008). "Safety evaluation of an oak-flavored milk powder containing ellagitannins upon oral administration in the rat." J Agric Food Chem 56(8): 2857-2865. |
| 1. Babova, O., et al. (2016). "Extraction of bilberry (Vaccinium myrtillus) antioxidants using supercritical/subcritical CO2 and ethanol as co-solvent." Journal of Supercritical Fluids 107: 358-363. |
| 1. Baek, Y. S., et al. (2015). "Flavonoids from Fragaria ananassa calyx and their antioxidant capacities." Journal of the Korean Society for Applied Biological Chemistry 58(6): 787-793. |
| 1. Basu, A., et al. (2014). "Freeze-Dried Strawberries Lower Serum Cholesterol and Lipid Peroxidation in Adults with Abdominal Adiposity and Elevated Serum Lipids." Journal of Nutrition 144(6): 830-837. |
| 1. Basu, A., et al. (2011). "Low-energy cranberry juice decreases lipid oxidation and increases plasma antioxidant capacity in women with metabolic syndrome." Nutrition Research 31(3): 190-196. |
| 1. Beaulieu, L. P., et al. (2010). "Inhibitory Effect of the Cree Traditional Medicine Wiishichimanaanh (Vaccinium vitis-idaea) on Advanced Glycation Endproduct Formation: Identification of Active Principles." Phytotherapy Research 24(5): 741-747. |
| 1. Bermudez-Soto, M. J. and F. A. Tomas-Barberan (2004). "Evaluation of commercial red fruit juice concentrates as ingredients for antioxidant functional juices." European Food Research and Technology 219(2): 133-141. |
| 1. Bhullar, K. S. and H. P. Rupasinghe (2015). "Antioxidant and cytoprotective properties of partridgeberry polyphenols." Food Chem 168: 595-605. |
| 1. Blanton, C., et al. (2015). "Probiotics Blunt the Anti-Hypertensive Effect of Blueberry Feeding in Hypertensive Rats without Altering Hippuric Acid Production." PLoS One 10(11): 14. |
| 1. Bordonaba, J. G., et al. (2011). "A new acetonitrile-free mobile phase for HPLC-DAD determination of individual anthocyanins in blackcurrant and strawberry fruits: A comparison and validation study." Food Chem 129(3): 1265-1273. |
| 1. Branning, C., et al. (2009). "Blueberry husks and multi-strain probiotics affect colonic fermentation in rats." Br J Nutr 101(6): 859-870. |
| 1. Brown, E. M., et al. (2012). "Persistence of anticancer activity in berry extracts after simulated gastrointestinal digestion and colonic fermentation." PLoS One 7(11): e49740. |
| 1. Brown, E. M., et al. (2014). "Comparison of in vivo and in vitro digestion on polyphenol composition in lingonberries: potential impact on colonic health." Biofactors 40(6): 611-623. |
| 1. Buchert, J., et al. (2005). "Effect of enzyme-aided pressing on anthocyanin yield and profiles in bilberry and blackcurrant juices." J Sci Food Agric 85(15): 2548-2556. |
| 1. Caruso, M. C., et al. (2015). "Improvement of Analytical Methods for the Determination of Polyphenolic Bioactive Compounds in Berry Fruits." Journal of Chemistry: 6. |
| 1. Chang, C. H., et al. (2017). "Photoprotective effects of cranberry juice and its various fractions against blue light-induced impairment in human retinal pigment epithelial cells." Pharm Biol 55(1): 571-580. |
| 1. Chiozzi, R. Z., et al. (2017). "Evaluation of column length and particle size effect on the untargeted profiling of a phytochemical mixture by using UHPLC coupled to high-resolution mass spectrometry." J Sep Sci 40(12): 2541-2557. |
| 1. Cisowski, W., et al. (1998). "Application of planar chromatography to the analysis of secondary metabolites in callus cultures of different plant species." Jpc-Journal of Planar Chromatography-Modern Tlc 11(6): 441-446. |
| 1. Cote, J., et al. (2010). "Analyzing cranberry bioactive compounds." Crit Rev Food Sci Nutr 50(9): 872-888. |
| 1. Crews, F., et al. (2006). "BHT blocks NF-kappaB activation and ethanol-induced brain damage." Alcohol Clin Exp Res 30(11): 1938-1949. |
| 1. Davies, H. V., et al. (2010). "Metabolome variability in crop plant species - When, where, how much and so what?" Regulatory Toxicology and Pharmacology 58(3): S54-S61. |
| 1. Del Bo, C., et al. (2010). "Anthocyanin Absorption, Metabolism, and Distribution from a Wild Blueberry-Enriched Diet (Vaccinium angustifolium) Is Affected by Diet Duration in the Sprague-Dawley Rat." J Agric Food Chem 58(4): 2491-2497. |
| 1. Detre, Z., et al. (1986). "Studies on vascular permeability in hypertension: action of anthocyanosides." Clin Physiol Biochem 4(2): 143-149. |
| 1. Die, J. V., et al. (2016). "Global patterns of protein abundance during the development of cold hardiness in blueberry." Environmental and Experimental Botany 124: 11-21. |
| 1. Duarte, W. F., et al. (2010). "Raspberry (Rubus idaeus L.) wine: Yeast selection, sensory evaluation and instrumental analysis of volatile and other compounds." Food Research International 43(9): 2303-2314. |
| 1. Dudonne, S., et al. (2016). "Potentiation of the bioavailability of blueberry phenolic compounds by co-ingested grape phenolic compounds in mice, revealed by targeted metabolomic profiling in plasma and feces." Food Funct 7(8): 3421-3430. |
| 1. D'Urso, G., et al. (2015). "Integrated mass spectrometric and multivariate data analysis approaches for the discrimination of organic and conventional strawberry (Fragaria ananassa Duch.) crops." Food Research International 77: 264-272. |
| 1. D'Urso, G., et al. (2018). "Combination of LC-MS based metabolomics and antioxidant activity for evaluation of bioactive compounds in Fragaria vesca leaves from Italy." Journal of Pharmaceutical and Biomedical Analysis 150: 233-240. |
| 1. Duthie, S. J., et al. (2006). "The effects of cranberry juice consumption on antioxidant status and biomarkers relating to heart disease and cancer in healthy human volunteers." Eur J Nutr 45(2): 113-122. |
| 1. Ely, C. B., et al. (1993). "DETERMINING DIURON, SIMAZINE, AND METHIOCARB RESIDUES IN HIGHBUSH BLUEBERRIES (VACCINIUM-CORYMBOSUM)." Hortscience 28(1): 33-35. |
| 1. Fait, A., et al. (2008). "Reconfiguration of the achene and receptacle metabolic networks during strawberry fruit development." Plant Physiol 148(2): 730-750. |
| 1. Fan, J. L., et al. (2012). "Flavonoid constituents and antioxidant capacity in flowers of different Zhongyuan tree penoy cultivars." Journal of Functional Foods 4(1): 147-157. |
| 1. Fang, X. L., et al. (2013). "Comparative Proteome Analysis of the Strawberry-Fusarium oxysporum f. sp fragariae Pathosystem Reveals Early Activation of Defense Responses as a Crucial Determinant of Host Resistance." J Proteome Res 12(4): 1772-1788. |
| 1. Feldman, M., et al. (2012). "Cranberry proanthocyanidins inhibit the adherence properties of Candida albicans and cytokine secretion by oral epithelial cells." BMC Complement Altern Med 12: 6. |
| 1. Feliciano, R. P., et al. (2015). "Methods to determine effects of cranberry proanthocyanidins on extraintestinal infections: Relevance for urinary tract health." Mol Nutr Food Res 59(7): 1292-1306. |
| 1. Fisher, D. R., et al. (2017). "Serum metabolites from walnut-fed aged rats attenuate stress-induced neurotoxicity in BV-2 microglial cells." Nutritional Neuroscience 20(2): 103-109. |
| 1. Grace, M. H., et al. (2009). "Hypoglycemic activity of a novel anthocyanin-rich formulation from lowbush blueberry, Vaccinium angustifolium Aiton." Phytomedicine 16(5): 406-415. |
| 1. Gras, C. C., et al. (2016). "Effect of genuine non-anthocyanin phenolics and chlorogenic acid on color and stability of black carrot (Daucus carota ssp sativus var. atrorubens Alef.) anthocyanins." Food Research International 85: 291-300. |
| 1. Grenier, J., et al. (2006). "Pomelo juice, but not cranberry juice, affects the pharmacokinetics of cyclosporine in humans." Clin Pharmacol Ther 79(3): 255-262. |
| 1. Gutierrez, E., et al. (2017). "Transcriptomics, Targeted Metabolomics and Gene Expression of Blackberry Leaves and Fruits Indicate Flavonoid Metabolic Flux from Leaf to Red Fruit." Frontiers in Plant Science 8: 15. |
| 1. Hanhineva, K. and A. Aharoni (2010). Metabolomics in Fruit Development. |
| 1. Hanhineva, K., et al. (2009). "Stilbene synthase gene transfer caused alterations in the phenylpropanoid metabolism of transgenic strawberry (Fragariaxananassa)." J Exp Bot 60(7): 2093-2106. |
| 1. Hanhineva, K., et al. (2008). "Non-targeted analysis of spatial metabolite composition in strawberry (Fragaria x ananassa) flowers." Phytochemistry 69(13): 2463-2481. |
| 1. Harris, C. S., et al. (2008). "Antidiabetic activity of extracts from needle, bark, and cone of Picea glauca: Organ-specific protection from glucose toxicity and glucose deprivation." Pharmaceutical Biology 46(1-2): 126-134. |
| 1. Hartl, K., et al. (2017). "Early metabolic and transcriptional variations in fruit of natural white-fruited Fragaria vesca genotypes." Sci Rep 7: 16. |
| 1. He, J., et al. (2006). "Intact anthocyanins and metabolites in rat urine and plasma after 3 months of anthocyanin supplementation." Nutr Cancer 54(1): 3-12. |
| 1. Heo, S., et al. (2011). "Metabolite fingerprinting of bokbunja (Rubus coreanus Miquel) by UPLC-qTOF-MS." Food Science and Biotechnology 20(2): 567-570. |
| 1. Hornedo-Ortega, R., et al. (2017). "Influence of Fermentation Process on the Anthocyanin Composition of Wine and Vinegar Elaborated from Strawberry." J Food Sci 82(2): 364-372. |
| 1. Hukkanen, A. T., et al. (2007). "Benzothiadiazole induces the accumulation of phenolics and improves resistance to powdery mildew in strawberries." J Agric Food Chem 55(5): 1862-1870. |
| 1. Hurst, R. D., et al. (2010). "Blueberry fruit polyphenolics suppress oxidative stress-induced skeletal muscle cell damage in vitro." Mol Nutr Food Res 54(3): 353-363. |
| 1. Ichiyanagi, T., et al. (2004). "Absorption and metabolism of delphinidin 3-O-beta-D-glucopyranoside in rats." Free Radical Biology and Medicine 36(7): 930-937. |
| 1. Ichiyanagi, T., et al. (2006). "Bioavailability and tissue distribution of anthocyanins in bilberry (Vaccinium myrtillus L.) extract in rats." J Agric Food Chem 54(18): 6578-6587. |
| 1. Jakesevic, M., et al. (2011). "Antioxidative protection of dietary bilberry, chokeberry and Lactobacillus plantarum HEAL19 in mice subjected to intestinal oxidative stress by ischemia-reperfusion." BMC Complement Altern Med 11: 12. |
| 1. Jones, A. G., et al. (2016). "The functional quality of decomposing litter outputs from an Arctic plant community is affected by long-term exposure to enhanced UV-B." Ecological Indicators 60: 8-17. |
| 1. Jooyandeh, H., et al. (2018). "Modeling of ultrasound-assisted extraction, characterization and in vitro pharmacological potential of polysaccharides from Vaccinium arctostaphylos L." Int J Biol Macromol 107: 938-948. |
| 1. Jurgonski, A., et al. (2017). "Metabolism of strawberry mono- and dimeric ellagitannins in rats fed a diet containing fructo-oligosaccharides." Eur J Nutr 56(2): 853-864. |
| 1. Jurica, K., et al. (2017). "Arbutin and its metabolite hydroquinone as the main factors in the antimicrobial effect of strawberry tree (Arbutus unedo L.) leaves." Journal of Herbal Medicine 8: 17-23. |
| 1. Jurica, K., et al. (2015). "Quantitative analysis of arbutin and hydroquinone in strawberry tree (Arbutus unedo L., Ericaceae) leaves by gas chromatography-mass spectrometry." Arhiv Za Higijenu Rada I Toksikologiju-Archives of Industrial Hygiene and Toxicology 66(3): 197-202. |
| 1. Kalt, W., et al. (2008). "Identification of anthocyanins in the liver, eye, and brain of blueberry-fed pigs." J Agric Food Chem 56(3): 705-712. |
| 1. Kalt, W., et al. (2010). "Recent Research on Polyphenolics in Vision and Eye Health." J Agric Food Chem 58(7): 4001-4007. |
| 1. Kapetanovic, I. M., et al. (2011). "Pharmacokinetics, oral bioavailability, and metabolic profile of resveratrol and its dimethylether analog, pterostilbene, in rats." Cancer Chemotherapy and Pharmacology 68(3): 593-601. |
| 1. Karppinen, K., et al. (2016). "Carotenoid metabolism during bilberry (Vaccinium myrtillus L.) fruit development under different light conditions is regulated by biosynthesis and degradation." BMC Plant Biol 16: 95. |
| 1. Khakimov, B., et al. (2013). "The use of trimethylsilyl cyanide derivatization for robust and broad-spectrum high-throughput gas chromatography-mass spectrometry based metabolomics." Anal Bioanal Chem 405(28): 9193-9205. |
| 1. Khanal, R., et al. (2014). "Urinary Excretion of Phenolic Acids in Rats Fed Cranberry, Blueberry, or Black Raspberry Powder." J Agric Food Chem 62(18): 3987-3996. |
| 1. Khanal, R. C., et al. (2010). "Urinary Excretion of (Epi)catechins in Rats Fed Different Berries or Berry Products." J Agric Food Chem 58(21): 11257-11264. |
| 1. Kim, D. S., et al. (2013). "Composition of Secondary Metabolites in Various Parts of 'Seolhyang' Strawberry Plants." Korean Journal of Horticultural Science & Technology 31(2): 224-230. |
| 1. Kim, H., et al. (2006). "Proteomics analysis of the actions of grape seed extract in rat brain: technological and biological implications for the study of the actions of psychoactive compounds." Life Sci 78(18): 2060-2065. |
| 1. Kim, M. S., et al. (2016). "Metabolic Response of Strawberry (Fragaria x ananassa) Leaves Exposed to the Angular Leaf Spot Bacterium (Xanthomonas fragariae)." J Agric Food Chem 64(9): 1889-1898. |
| 1. Koehler, G., et al. (2012). "Proteomic study of low-temperature responses in strawberry cultivars (Fragaria x ananassa) that differ in cold tolerance." Plant Physiol 159(4): 1787-1805. |
| 1. Koh, T. H. and L. D. Melton (2002). "Ripening-related changes in cell wall polysaccharides of strawberry cortical and pith tissues." Postharvest Biology and Technology 26(1): 23-33. |
| 1. Koo, H., et al. (2010). "Influence of cranberry proanthocyanidins on formation of biofilms by Streptococcus mutans on saliva-coated apatitic surface and on dental caries development in vivo." Caries Res 44(2): 116-126. |
| 1. Koskimaki, J. J., et al. (2009). "Flavonoid biosynthesis and degradation play a role in early defence responses of bilberry (Vaccinium myrtillus) against biotic stress." European Journal of Plant Pathology 125(4): 629-640. |
| 1. Kosmala, M., et al. (2014). "Chemical composition of polyphenols extracted from strawberry pomace and their effect on physiological properties of diets supplemented with different types of dietary fibre in rats." Eur J Nutr 53(2): 521-532. |
| 1. Lala, G., et al. (2006). "Anthocyanin-rich extracts inhibit multiple biomarkers of colon cancer in rats." Nutr Cancer 54(1): 84-93. |
| 1. Lavola, A., et al. (2017). "Phytochemical variation in the plant-part specific phenols of wild crowberry (Empetrum hermaphroditum Hagerup) populations." Phytochemistry Letters 21: 11-20. |
| 1. Lehtonen, H. M., et al. (2010). "Flavonol glycosides of sea buckthorn (Hippophae rhamnoides ssp. sinensis) and lingonberry (Vaccinium vitis-idaea) are bioavailable in humans and monoglucuronidated for excretion." J Agric Food Chem 58(1): 620-627. |
| 1. Lehtonen, H. M., et al. (2013). "H-1 NMR-based metabolic fingerprinting of urine metabolites after consumption of lingonberries (Vaccinium vitis-idaea) with a high-fat meal." Food Chem 138(2-3): 982-990. |
| 1. Lehtonen, H. M., et al. (2009). "Urinary Excretion of the Main Anthocyanin in Lingonberry (Vaccinium vitis-idaea), Cyanidin 3-O-Galactoside, and Its Metabolites." J Agric Food Chem 57(10): 4447-4451. |
| 1. Li, C., et al. (2013). "Oxygen radical absorbance capacity of different varieties of strawberry and the antioxidant stability in storage." Molecules 18(2): 1528-1539. |
| 1. Li, L., et al. (2013). "Quantitative proteomic investigation employing stable isotope labeling by peptide dimethylation on proteins of strawberry fruit at different ripening stages." Journal of Proteomics 94: 219-239. |
| 1. Li, L. L., et al. (2016). "Comparative transcriptome sequencing and de novo analysis of Vaccinium corymbosum during fruit and color development." Bmc Plant Biology 16: 9. |
| 1. Lin, X., et al. (2013). "The effect of RNAi-induced silencing of FaDFR on anthocyanin metabolism in strawberry (Fragaria x ananassa) fruit." Scientia Horticulturae 160: 123-128. |
| 1. Lingua, G., et al. (2013). "Arbuscular Mycorrhizal Fungi and Plant Growth-Promoting Pseudomonads Increases Anthocyanin Concentration in Strawberry Fruits (Fragaria x ananassa var. Selva) in Conditions of Reduced Fertilization." Int J Mol Sci 14(8): 16207-16225. |
| 1. Liu, C. J. and J. Y. Lin (2013). "Anti-inflammatory effects of phenolic extracts from strawberry and mulberry fruits on cytokine secretion profiles using mouse primary splenocytes and peritoneal macrophages." International Immunopharmacology 16(2): 165-170. |
| 1. Liu, P. Z., et al. (2014). "Characterization of Metabolite Profiles of Leaves of Bilberry (Vaccinium myrtillus L.) and Lingonberry (Vaccinium vitis-idaea L.)." J Agric Food Chem 62(49): 12015-12026. |
| 1. Liu, S., et al. (2017). "Comparative transcriptomic analysis of key genes involved in flavonoid biosynthetic pathway and identification of a flavonol synthase from Artemisia annua L." Plant Biology 19(4): 618-629. |
| 1. Liu, Y. X., et al. (2011). "Inhibitory Effect of Blueberry Polyphenolic Compounds on Oleic Acid-Induced Hepatic Steatosis in Vitro." J Agric Food Chem 59(22): 12254-12263. |
| 1. Lowenthal, M. S., et al. (2013). "Characterizing Vaccinium berry Standard Reference Materials by GCaEuroMS using NIST spectral libraries." Anal Bioanal Chem 405(13): 4467-4476. |
| 1. Madrigal-Carballo, S., et al. (2009). "Chitosomes loaded with cranberry proanthocyanidins attenuate the bacterial lipopolysaccharide-induced expression of iNOS and COX-2 in raw 264.7 macrophages." J Liposome Res 19(3): 189-196. |
| 1. Males, Z., et al. (2013). "Quantitative Determination of Flavonoids and Chlorogenic Acid in the Leaves of Arbutus unedo L. Using Thin Layer Chromatography." Journal of Analytical Methods in Chemistry: 4. |
| 1. Mamani, A., et al. (2012). "Pathogen-Induced Accumulation of an Ellagitannin Elicits Plant Defense Response." Molecular Plant-Microbe Interactions 25(11): 1430-1439. |
| 1. Mateos, R., et al. (2005). "Determination of malondialdehyde (MDA) by high-performance liquid chromatography in serum and liver as a biomarker for oxidative stress. Application to a rat model for hypercholesterolemia and evaluation of the effect of diets rich in phenolic antioxidants from fruits." J Chromatogr B Analyt Technol Biomed Life Sci 827(1): 76-82. |
| 1. Mathison, B. D., et al. (2014). "Consumption of cranberry beverage improved endogenous antioxidant status and protected against bacteria adhesion in healthy humans: a randomized controlled trial." Nutr Res 34(5): 420-427. |
| 1. Matsumoto, H., et al. (2006). "Comparative assessment of distribution of blackcurrant anthocyanins in rabbit and rat ocular tissues." Experimental Eye Research 83(2): 348-356. |
| 1. Matsunaga, N., et al. (2009). "Bilberry and its main constituents have neuroprotective effects against retinal neuronal damage in vitro and in vivo." Mol Nutr Food Res 53(7): 869-877. |
| 1. McKay, D. L., et al. (2015). "Flavonoids and phenolic acids from cranberry juice are bioavailable and bioactive in healthy older adults." Food Chem 168: 233-240. |
| 1. Medina-Puche, L., et al. (2015). "An R2R3-MYB Transcription Factor Regulates Eugenol Production in Ripe Strawberry Fruit Receptacles." Plant Physiol 168(2): 598-614. |
| 1. Milala, J., et al. (2017). "Ellagitannins from Strawberries with Different Degrees of Polymerization Showed Different Metabolism through Gastrointestinal Tract of Rats." J Agric Food Chem 65(49): 10738-10748. |
| 1. Milbury, P. E., et al. (2007). "Bilberry (Vaccinium myrtillus) anthocyanins modulate heme oxygenase-1 and glutathione S-transferase-pi expression in ARPE-19 cells." Invest Ophthalmol Vis Sci 48(5): 2343-2349. |
| 1. Miret, J. A., et al. (2014). "Application of a Rapid and Sensitive Method for Hormonal and Vitamin E Profiling Reveals Crucial Regulatory Mechanisms in Flower Senescence and Fruit Ripening." Journal of Plant Growth Regulation 33(1): 34-43. |
| 1. Monschein, M., et al. (2015). "Content of phenolic compounds in wild populations of Epilobium angustifolium growing at different altitudes." Pharmaceutical Biology 53(11): 1576-1582. |
| 1. Morazzoni, P., et al. (1991). "Vaccinium myrtillus anthocyanosides pharmacokinetics in rats." Arzneimittelforschung 41(2): 128-131. |
| 1. Muller-Maatsch, J., et al. (2016). "Co-pigmentation of pelargonidin derivatives in strawberry and red radish model solutions by the addition of phenolic fractions from mango peels." Food Chem 213: 625-634. |
| 1. Munoz, C., et al. (2011). "Polyphenol Composition in the Ripe Fruits of Fragaria Species and Transcriptional Analyses of Key Genes in the Pathway." J Agric Food Chem 59(23): 12598-12604. |
| 1. Navarro, M., et al. (2017). "Fractioning of Proanthocyanidins of Uncaria tomentosa. Composition and Structure-Bioactivity Relationship." Antioxidants 6(3): 13. |
| 1. Nieman, D. C., et al. (2013). "Influence of a Polyphenol-Enriched Protein Powder on Exercise-Induced Inflammation and Oxidative Stress in Athletes: A Randomized Trial Using a Metabolomics Approach." PLoS One 8(8): 11. |
| 1. Ohnishi, R., et al. (2006). "Urinary excretion of anthocyanins in humans after cranberry juice ingestion." Bioscience Biotechnology and Biochemistry 70(7): 1681-1687. |
| 1. Oliveira, P. S., et al. (2017). "Vaccinium virgatum fruit extract as an important adjuvant in biochemical and behavioral alterations observed in animal model of metabolic syndrome." Biomed Pharmacother 88: 939-947. |
| 1. Olsson, M. E., et al. (2006). "Antioxidant levels and inhibition of cancer cell proliferation in vitro by extracts from organically and conventionally cultivated strawberries." J Agric Food Chem 54(4): 1248-1255. |
| 1. Osorio, S., et al. (2011). "Demethylation of oligogalacturonides by FaPE1 in the fruits of the wild strawberry Fragaria vesca triggers metabolic and transcriptional changes associated with defence and development of the fruit." J Exp Bot 62(8): 2855-2873. |
| 1. Oszmianski, J., et al. (2011). "Identification and Characterization of Low Molecular Weight Polyphenols in Berry Leaf Extracts by HPLC-DAD and LC-ESI/MS." J Agric Food Chem 59(24): 12830-12835. |
| 1. Paolocci, F., et al. (2011). "The strawberry transcription factor FaMYB1 inhibits the biosynthesis of proanthocyanidins in Lotus corniculatus leaves." J Exp Bot 62(3): 1189-1200. |
| 1. Paquette, M., et al. (2017). "Strawberry and cranberry polyphenols improve insulin sensitivity in insulin-resistant, non-diabetic adults: a parallel, double-blind, controlled and randomised clinical trial." Br J Nutr 117(4): 519-531. |
| 1. Paudel, L., et al. (2013). "Nonanthocyanin Secondary Metabolites of Black Raspberry (Rubus occidentalis L.) Fruits: Identification by HPLC-DAD, NMR, HPLC-ESI-MS, and ESI-MS/MS Analyses." J Agric Food Chem 61(49): 12032-12043. |
| 1. Paunovic, S. M., et al. (2017). "Bioactive compounds and antimicrobial activity of black currant (Ribes nigrum L.) berries and leaves extract obtained by different soil management system." Scientia Horticulturae 222: 69-75. |
| 1. Pawlaczyk, I., et al. (2009). "Polyphenolic-polysaccharide compounds from selected medicinal plants of Asteraceae and Rosaceae families: Chemical characterization and blood anticoagulant activity." Carbohydr Polym 77(3): 568-575. |
| 1. Peron, G., et al. (2017). "The antiadhesive activity of cranberry phytocomplex studied by metabolomics: Intestinal PAC-A metabolites but not intact PAC-A are identified as markers in active urines against uropathogenic Escherichia coil." Fitoterapia 122: 67-75. |
| 1. Peron, G., et al. (2017). "The antiadhesive activity of cranberry phytocomplex studied by metabolomics: Intestinal PAC-A metabolites but not intact PAC-A are identified as markers in active urines against uropathogenic Escherichia coli." Fitoterapia 122: 67-75. |
| 1. Popovic, Z., et al. (2018). "Geographic variability of selected phenolic compounds in fresh berries of two Cornus species." Trees-Structure and Function 32(1): 203-214. |
| 1. Prinsi, B., et al. (2016). "Proteomic Comparison of Fruit Ripening between 'Hedelfinger' Sweet Cherry (Prunus avium L.) and Its Somaclonal Variant 'HS'." J Agric Food Chem 64(20): 4171-4181. |
| 1. Rashid, J., et al. (1993). "Quercetin, an in vitro inhibitor of CYP3A, does not contribute to the interaction between nifedipine and grapefruit juice." Br J Clin Pharmacol 36(5): 460-463. |
| 1. Rebolledo, R., et al. (2012). "The effects of the essential oil and hydrolate of canelo (Drimys winteri) on adults of Aegorhinus superciliosus in the laboratory." Ciencia E Investigacion Agraria 39(3): 481-488. |
| 1. Ribera, A., et al. (2008). "Effect of extracts from in vitro-grown shoots of Quillaja saponaria Mol. on Botrytis cinerea Pers." World J Microbiol Biotechnol 24(9): 1803-1811. |
| 1. Rieger, G., et al. (2008). "Influence of altitudinal variation on the content of phenolic compounds in wild populations of Calluna vulgaris, Sambucus nigra, and Vaccinium myrtillus." J Agric Food Chem 56(19): 9080-9086. |
| 1. Ring, L., et al. (2013). "Metabolic Interaction between Anthocyanin and Lignin Biosynthesis Is Associated with Peroxidase FaPRX27 in Strawberry Fruit." Plant Physiol 163(1): 43-60. |
| 1. Rodriguez-Mateos, A., et al. (2014). "Impact of processing on the bioavailability and vascular effects of blueberry (poly)phenols." Mol Nutr Food Res 58(10): 1952-1961. |
| 1. Roopchand, D. E., et al. (2012). "Efficient sorption of polyphenols to soybean flour enables natural fortification of foods." Food Chem 131(4): 1193-1200. |
| 1. Ruel, G., et al. (2013). "Evidence that cranberry juice may improve augmentation index in overweight men." Nutr Res 33(1): 41-49. |
| 1. Sakakibara, H., et al. (2009). "Distribution and Excretion of Bilberry Anthocyanines in Mice." J Agric Food Chem 57(17): 7681-7686. |
| 1. Salaheen, S., et al. (2017). "Alternative Growth Promoters Modulate Broiler Gut Microbiome and Enhance Body Weight Gain." Frontiers in Microbiology 8: 11. |
| 1. Saleem, A., et al. (2010). "A RP-HPLC-DAD-APCl/MSD Method for the Characterisation of Medicinal Ericaceae used by the Eeyou Istchee Cree First Nations." Phytochemical Analysis 21(4): 328-339. |
| 1. Sanchez-Patan, F., et al. (2012). "Comprehensive Assessment of the Quality of Commercial Cranberry Products. Phenolic Characterization and in Vitro Bioactivity." J Agric Food Chem 60(13): 3396-3408. |
| 1. Seker, M. and C. Toplu (2010). "Determination and Comparison of Chemical Characteristics of Arbutus unedo L. and Arbutus andrachnae L. (Family Ericaceae) Fruits." J Med Food 13(4): 1013-1018. |
| 1. Selma, M. V., et al. (2017). "Isolation of Human Intestinal Bacteria Capable of Producing the Bioactive Metabolite Isourolithin A from Ellagic Acid." Frontiers in Microbiology 8: 8. |
| 1. Silva, B. M., et al. (2011). "Dracaena draco L. fruit: Phytochemical and antioxidant activity assessment." Food Research International 44(7): 2182-2189. |
| 1. Sinelli, N., et al. (2011). "Near infrared (NIR) spectroscopy as a tool for monitoring blueberry osmo-air dehydration process." Food Research International 44(5): 1427-1433. |
| 1. Song, C., et al. (2015). "Functional Characterization and Substrate Promiscuity of UGT71 Glycosyltransferases from Strawberry (Fragaria x ananassa)." Plant Cell Physiol 56(12): 2478-2493. |
| 1. Stoner, G. D., et al. (2006). "Protection against esophageal cancer in rodents with lyophilized berries: Potential mechanisms." Nutrition and Cancer-an International Journal 54(1): 33-46. |
| 1. Student, V., et al. (2016). "Cranberry intervention in patients with prostate cancer prior to radical prostatectomy. Clinical, pathological and laboratory findings." Biomedical Papers-Olomouc 160(4): 559-565. |
| 1. Sun, H., et al. (2015). "De novo sequencing and analysis of the cranberry fruit transcriptome to identify putative genes involved in flavonoid biosynthesis, transport and regulation." Bmc Genomics 16: 652. |
| 1. Takacs, I., et al. (2017). "HPLC METHOD FOR MEASUREMENT OF HUMAN SALIVARY alpha-AMYLASE INHIBITION BY AQUEOUS PLANT EXTRACTS." Acta Biologica Hungarica 68(2): 127-136. |
| 1. Talavera, S., et al. (2005). "Anthocyanin metabolism in rats and their distribution to digestive area, kidney, and brain." J Agric Food Chem 53(10): 3902-3908. |
| 1. Talavera, S., et al. (2004). "Anthocyanins are efficiently absorbed from the small intestine in rats." J Nutr 134(9): 2275-2279. |
| 1. Terlizzi, M. E., et al. (2016). "Inhibition of herpes simplex type 1 and type 2 infections by Oximacro((R)), a cranberry extract with a high content of A-type proanthocyanidins (PACs-A)." Antiviral Res 132: 154-164. |
| 1. Timmers, M. A., et al. (2017). "Inter- and intra-seasonal changes in anthocyanin accumulation and global metabolite profiling of six blueberry genotypes." Journal of Food Composition and Analysis 59: 105-110. |
| 1. Torronen, R., et al. (2012). "Postprandial glucose, insulin, and free fatty acid responses to sucrose consumed with blackcurrants and lingonberries in healthy women." American Journal of Clinical Nutrition 96(3): 527-533. |
| 1. Torronen, R., et al. (2012). "Fortification of blackcurrant juice with crowberry: Impact on polyphenol composition, urinary phenolic metabolites, and postprandial glycemic response in healthy subjects." Journal of Functional Foods 4(4): 746-756. |
| 1. Trost, K., et al. (2008). "Anthocyanin degradation of blueberry-aronia nectar in glass compared with carton during storage." J Food Sci 73(8): S405-411. |
| 1. Tuberoso, C. I. G., et al. (2016). "Antioxidant activity, cytotoxic activity and metabolic profiling of juices obtained from saffron (Crocus sativus L.) floral by-products." Food Chem 199: 18-27. |
| 1. Tulio, A. Z., et al. (2012). "Berry Fruits Modulated Endothelial Cell Migration and Angiogenesis via Phosphoinositide-3 Kinase/Protein Kinase B Pathway in Vitro in Endothelial Cells." J Agric Food Chem 60(23): 5803-5812. |
| 1. Tzortzakis, N., et al. (2007). "Impact of atmospheric ozone-enrichment on quality-related attributes of tomato fruit." Postharvest Biology and Technology 45(3): 317-325. |
| 1. Uberos, J., et al. (2015). "Phenolic acid content and antiadherence activity in the urine of patients treated with cranberry syrup (Vaccinium macrocarpon) vs. trimethoprim for recurrent urinary tract infection." Journal of Functional Foods 18: 608-616. |
| 1. Uleberg, E., et al. (2012). "Effects of Temperature and Photoperiod on Yield and Chemical Composition of Northern and Southern Clones of Bilberry (Vaccinium myrtillus L.)." J Agric Food Chem 60(42): 10406-10414. |
| 1. Urrutia, M., et al. (2016). "Genetic dissection of the (poly)phenol profile of diploid strawberry (Fragaria vesca) fruits using a NIL collection." Plant Science 242: 151-168. |
| 1. van Dooren, I., et al. (2018). "Advantages of a validated UPLC-MS/MS standard addition method for the quantification of A-type dimeric and trimeric proanthocyanidins in cranberry extracts in comparison with well-known quantification methods." Journal of Pharmaceutical and Biomedical Analysis 148: 32-41. |
| 1. Vostalova, J., et al. (2015). "Are High Proanthocyanidins Key to Cranberry Efficacy in the Prevention of Recurrent Urinary Tract Infection?" Phytotherapy Research 29(10): 1559-1567. |
| 1. Vvedenskaya, I. O., et al. (2004). "Characterization of flavonols in cranberry (Vaccinium macrocarpon) powder." J Agric Food Chem 52(2): 188-195. |
| 1. Walsh, J. M., et al. (2016). "Liquid chromatography with tandem mass spectrometry quantification of urinary proanthocyanin A2 dimer and its potential use as a biomarker of cranberry intake." J Sep Sci 39(2): 342-349. |
| 1. Wang, S. Y., et al. (2007). "Antioxidant activity of Vaccinium stamineum: exhibition of anticancer capability in human lung and leukemia cells." Planta Med 73(5): 451-460. |
| 1. Witzell, J., et al. (2003). "Plant-part specific and temporal variation in phenolic compounds of boreal bilberry (Vaccinium myrtillus) plants." Biochemical Systematics and Ecology 31(2): 115-127. |
| 1. Witzell, J. and A. Shevtsova (2004). "Nitrogen-induced changes in phenolics of Vaccinium myrtillus - Implications for interaction with a parasitic fungus." J Chem Ecol 30(10): 1937-1956. |
| 1. Zhu, L., et al. (2013). "Influence of harvest season on antioxidant activity and constituents of rabbiteye blueberry ( Vaccinium ashei ) leaves." J Agric Food Chem 61(47): 11477-11483. |
| 1. Zhu, L. C., et al. (2013). "Influence of Harvest Season on Antioxidant Activity and Constituents of Rabbiteye Blueberry (Vaccinium ashei) Leaves." J Agric Food Chem 61(47): 11477-11483. |
| 1. Zhu, X. R., et al. (2015). "Lyophilized strawberries prevent 7,12-dimethylbenz alpha anthracene (DMBA)-induced oral squamous cell carcinogenesis in hamsters." Journal of Functional Foods 15: 476-486. |
| 1. Ziberna, L., et al. (2010). "Acute Cardioprotective and Cardiotoxic Effects of Bilberry Anthocyanins in Ischemia-Reperfusion Injury: Beyond Concentration-Dependent Antioxidant Activity." Cardiovascular Toxicology 10(4): 283-294. |
| 1. Zoratti, L., et al. (2014). "Monochromatic light increases anthocyanin content during fruit development in bilberry." Bmc Plant Biology 14: 10. |
| 1. Zorenc, Z., et al. (2016). "White versus blue: Does the wild 'albino' bilberry (Vaccinium myrtillus L.) differ in fruit quality compared to the blue one?" Food Chem 211: 876-882. |
| 1. Zuniga, G. E., et al. (2012). "Effect of ionizing energy on extracts of Quillaja saponaria to be used as an antimicrobial agent on irradiated edible coating for fresh strawberries." Radiation Physics and Chemistry 81(1): 64-69. |
