# Supplementary material for: Contribution of Berry Polyphenols to the Human Metabolome
Source: Molecules. 2019 Nov 20;24(23):4220. doi: 10.3390/molecules24234220 (PMC6930569; doi:10.3390/molecules24234220)
Supplement: Supplementary file 1 [file molecules-24-04220-s001.zip › Supplement 4.docx]

**S4. Excluded references at abstract evaluation stage**

| 1. Antonio, B., et al. (2017). "Real-time monitoring of glucose and phenols intestinal absorption through an integrated Caco-2TC7cells/biosensors telemetric device: Hypoglycemic effect of fruit phytochemicals." Biosensors & Bioelectronics 88: 159-166. |
| --- |
| 1. Bialasiewicz, P., et al. (2014). "Addition of strawberries to the usual diet decreases resting chemiluminescence of fasting blood in healthy subjects-possible health-promoting effect of these fruits consumption." J Am Coll Nutr 33(4): 274-287. |
| 1. Cheel, J., et al. (2005). "E-cinnamic acid derivatives and phenolics from Chilean strawberry fruits, Fragaria chiloensis ssp. chiloensis." J Agric Food Chem 53(22): 8512-8518. |
| 1. Clegg, M. E., et al. (2011). "The addition of raspberries and blueberries to a starch-based food does not alter the glycaemic response." Br J Nutr 106(3): 335-338. |
| 1. Crecelius, A. C., et al. (2017). "Spatial and Temporal Localization of Flavonoid Metabolites in Strawberry Fruit (Fragaria x ananassa)." J Agric Food Chem 65(17): 3559-3568. |
| 1. de Mello, V. D. F., et al. (2017). "Fasting serum hippuric acid is elevated after bilberry (Vaccinium myrtillus) consumption and associates with improvement of fasting glucose levels and insulin secretion in persons at high risk of developing type 2 diabetes." Mol Nutr Food Res 61(9): 8. |
| 1. Del Bo, C., et al. (2017). "A serving of blueberry (V. corymbosum) acutely improves peripheral arterial dysfunction in young smokers and non-smokers: two randomized, controlled, crossover pilot studies." Food Funct 8(11): 4108-4117. |
| 1. Del Bo, C., et al. (2014). "A single serving of blueberry (V-corymbosum) modulates peripheral arterial dysfunction induced by acute cigarette smoking in young volunteers: a randomized-controlled trial." Food Funct 5(12): 3107-3116. |
| 1. Del Bo, C., et al. (2013). "A single portion of blueberry (Vaccinium corymbosum L) improves protection against DNA damage but not vascular function in healthy male volunteers." Nutr Res 33(3): 220-227. |
| 1. D'Urso, G., et al. (2016). "Characterisation of Fragaria vesca fruit from Italy following a metabolomics approach through integrated mass spectrometry techniques." Lwt-Food Science and Technology 74: 387-395. |
| 1. Erlund, I., et al. (2003). "Consumption of black currants, lingonberries and bilberries increases serum quercetin concentrations." Eur J Clin Nutr 57(1): 37-42. |
| 1. Espin, J. C., et al. (2007). "Iberian pig as a model to clarify obscure points in the bioavailability and metabolism of ellagitannins in humans." J Agric Food Chem 55(25): 10476-10485. |
| 1. Faria, A., et al. (2005). "Antioxidant properties of prepared blueberry (Vaccinium myrtillus) extracts." J Agric Food Chem 53(17): 6896-6902. |
| 1. Felgines, C., et al. (2007). "Strawberry pelargonidin glycosides are excreted in urine as intact glycosides and glucuronidated pelargonidin derivatives in rats." Br J Nutr 98(6): 1126-1131. |
| 1. Feliciano, R. P., et al. (2016). "Plasma and Urinary Phenolic Profiles after Acute and Repetitive Intake of Wild Blueberry." Molecules 21(9): 15. |
| 1. Feliciano, R. P., et al. (2017). "Absorption, Metabolism and Excretion of Cranberry (Poly)phenols in Humans: A Dose Response Study and Assessment of Inter-Individual Variability." Nutrients 9(3). |
| 1. Gizzi, C., et al. (2016). "Bilberry extracts are not created equal: the role of non anthocyanin fraction. Discovering the "dark side of the force" in a preliminary study." European Review for Medical and Pharmacological Sciences 20(11): 2418-2424. |
| 1. Griesser, M., et al. (2008). "Multi-substrate flavonol O-glucosyltransferases from strawberry (Fragariaxananassa) achene and receptacle." J Exp Bot 59(10): 2611-2625. |
| 1. Halbwirth, H., et al. (2006). "Two-phase flavonoid formation in developing strawberry (Fragaria x ananassa) fruit." J Agric Food Chem 54(4): 1479-1485. |
| 1. Harris, C. S., et al. (2007). "A single HPLC-PAD-APCI/MS method for the quantitative comparison of phenolic compounds found in leaf, stem, root and fruit extracts of Vaccinium angustifolium." Phytochemical Analysis 18(2): 161-169. |
| 1. Iswaldi, I., et al. (2013). "Identification of polyphenols and their metabolites in human urine after cranberry-syrup consumption." Food and Chemical Toxicology 55: 484-492. |
| 1. Jovancevic, M., et al. (2011). "Analysis of phenolic compounds in wild populations of bilberry (Vaccinium myrtillus L.) from Montenegro." Journal of Medicinal Plants Research 5(6): 910-914. |
| 1. Kellogg, J., et al. (2010). "Alaskan Wild Berry Resources and Human Health under the Cloud of Climate Change." J Agric Food Chem 58(7): 3884-3900. |
| 1. Kim, D. Y., et al. (2015). "Differentiation of highbush blueberry (Vaccinium corymbosum L.) fruit cultivars by GC-MS-based metabolic profiling." Journal of the Korean Society for Applied Biological Chemistry 58(1): 21-28. |
| 1. Kolehmainen, M., et al. (2012). "Bilberries reduce low-grade inflammation in individuals with features of metabolic syndrome." Mol Nutr Food Res 56(10): 1501-1510. |
| 1. Koponen, J. M., et al. (2008). "Characterization and fate of black currant and bilberry flavonols in enzyme-aided processing." J Agric Food Chem 56(9): 3136-3144. |
| 1. Kosinska, A., et al. (2012). "Stability of Phenolic Compounds Isolated from Cocoa, Green Tea and Strawberries in Hank's Balanced Salt Solution under Cell Culture Conditions." Polish Journal of Food and Nutrition Sciences 62(2): 91-96. |
| 1. McIntyre, K. L., et al. (2009). "Seasonal Phytochemical Variation of Anti-Glycation Principles in Lowbush Blueberry (Vaccinium angustifolium)." Planta Med 75(3): 286-292. |
| 1. Molan, A. L., et al. (2009). "In vitro and in vivo evaluation of the prebiotic activity of water-soluble blueberry extracts." World J Microbiol Biotechnol 25(7): 1243-1249. |
| 1. Nagulsamy, P., et al. (2015). "Evaluation of antioxidant, anti-inflammatory, and antiulcer properties of Vaccinium leschenaultii Wight: A therapeutic supplement." J Food Drug Anal 23(3): 376-386. |
| 1. Najda, A., et al. (2014). "Comparative analysis of secondary metabolites contents in Fragaria vesca L. fruits." Annals of Agricultural and Environmental Medicine 21(2): 339-343. |
| 1. Ranger, C. M., et al. (2007). "Intraspecific variation in aphid resistance and constitutive phenolics exhibited by the wild blueberry Vaccinium darrowi." J Chem Ecol 33(4): 711-729. |
| 1. Riso, P., et al. (2013). "Effect of a wild blueberry (Vaccinium angustifolium) drink intervention on markers of oxidative stress, inflammation and endothelial function in humans with cardiovascular risk factors." Eur J Nutr 52(3): 949-961. |
| 1. Sanchez-Villavicencio, M. L., et al. (2017). "Fermented blueberry juice extract and its specific fractions have an anti-adipogenic effect in 3 T3-L1 cells." BMC Complement Altern Med 17: 9. |
| 1. Santhakumar, A. B., et al. (2015). "The ex vivo antiplatelet activation potential of fruit phenolic metabolite hippuric acid." Food Funct 6(8): 2679-2683. |
| 1. Simirgiotis, M. J. and G. Schmeda-Hirschmann (2010). "Determination of phenolic composition and antioxidant activity in fruits, rhizomes and leaves of the white strawberry (Fragaria chiloensis spp. chiloensis form chiloensis) using HPLC-DAD-ESI-MS and free radical quenching techniques." Journal of Food Composition and Analysis 23(6): 545-553. |
| 1. Sun, J. H., et al. (2014). "Profiling polyphenols of two diploid strawberry (Fragaria vesca) inbred lines using UHPLC-HRMSn." Food Chem 146: 289-298. |
| 1. Vendrame, S., et al. (2011). "Six-Week Consumption of a Wild Blueberry Powder Drink Increases Bifidobacteria in the Human Gut." J Agric Food Chem 59(24): 12815-12820. |
| 1. Zhang, J., et al. (2014). "Isolation and identification of antioxidant compounds in Vaccinium bracteatum Thunb. by UHPLC-Q-TOF LC/MS and their kidney damage protection." Journal of Functional Foods 11: 62-70. |
| 1. Zhao, Y., et al. (2017). "Retention of polyphenols in blueberries (Vaccinium corymbosum) after different cooking methods, using UHPLC-DAD-MS based metabolomics." Journal of Food Composition and Analysis 56: 55-66. |
| 1. Zifkin, M., et al. (2012). "Gene Expression and Metabolite Profiling of Developing Highbush Blueberry Fruit Indicates Transcriptional Regulation of Flavonoid Metabolism and Activation of Abscisic Acid Metabolism." Plant Physiol 158(1): 200-224. |
