# Supplementary material for: Contribution of Berry Polyphenols to the Human Metabolome
Source: Molecules. 2019 Nov 20;24(23):4220. doi: 10.3390/molecules24234220 (PMC6930569; doi:10.3390/molecules24234220)
Supplement: Supplementary file 1 [file molecules-24-04220-s001.zip › Supplement 5.docx]

**S5. Excluded references at full text evaluation stage**

| 1. Burton-Freeman, B., et al., Strawberry modulates LDL oxidation and postprandial lipemia in response to high-fat meal in overweight hyperlipidemic men and women. J Am Coll Nutr, 2010. 29(1): p. 46-54 |
| --- |
| 1. Cebeci, F. and N. Sahin-Yesilcubuk, The matrix effect of blueberry, oat meal and milk on polyphenols, antioxidant activity and potential bioavailability. Int J Food Sci Nutr, 2014. 65(1): p. 69-78. |
| 1. Pineli, L.D.D., C.L. Moretti, and M.D. Chiarello, Quality, bioactive compounds and antioxidant activity of strawberries grown in the Brazilian savannah and stored at different temperatures. Journal of Food Agriculture & Environment, 2012. 10(2): p. 165-171. |
| 1. Zasowska-Nowak, A., et al., Strawberries Added to the Usual Diet Suppress Fasting Plasma Paraoxonase Activity and Have a Weak Transient Decreasing Effect on Cholesterol Levels in Healthy Nonobese Subjects. Journal of the American College of Nutrition, 2016. 35(5): p. 422-435. |
| 1. Alvarez-Fernandez, M.A., et al., Composition of nonanthocyanin polyphenols in alcoholic-fermented strawberry products using LC-MS (QTRAP), high-resolution MS (UHPLC-Orbitrap-MS), LC-DAD, and antioxidant activityfermented strawberry products using LC-MS (QTRAP), high-resolution MS (UHPLC-Orbitrap-MS), LC-DAD, and antioxidant activity |
| 1. Basu, A., et al., Blueberries Decrease Cardiovascular Risk Factors in Obese Men and Women with Metabolic Syndrome |
| 1. Basu, A., et al., Freeze-dried strawberries lower serum cholesterol and lipid peroxidation in adults with abdominal adiposity and elevated serum lipids |
| 1. Basu, A., et al., Freeze-dried strawberry powder improves lipid profile and lipid peroxidation in women with metabolic syndrome: |
| 1. Capocasa, F., et al., Breeding strawberry (Fragaria X ananassa Duch) to increase fruit nutritional quality. |
| 1. Cheatham, C.L., et al., Blueberry Consumption Affects Serum Uric Acid Concentrations in Older Adults in a Sex-Specific Manner |
| 1. Dymerski, T., et al., Chemistry and biological properties of berry volatiles by two-dimensional chromatography, fluorescence and Fourier transform infrared spectroscopy techniques |
| 1. Jungfer, E., et al., Comparing procyanidins in selected Vaccinium species by UHPLC-MS(2) with regard to authenticity and health effects |
| 1. McDougall, G., I. Martinussen, and D. Stewart, Towards fruitful metabolomics: high throughput analyses of polyphenol composition in berries using direct infusion mass spectrometry. J Chromatogr B Analyt Technol Biomed Life Sci, 2008. 871(2): p. 362-9. |
| 1. McDougall, G.J., N.N. Kulkarni, and D. Stewart, Berry polyphenols inhibit pancreatic lipase activity in vitro. Food Chemistry, 2009. 115(1): p. 193-199. |
| 1. Milbury, P.E. and W. Kalt, Xenobiotic Metabolism and Berry Flavonoid Transport across the Blood-Brain Barrier. Journal of Agricultural and Food Chemistry, 2010. 58(7): p. 3950-3956. |
| 1. Nurmi, T., et al., Metabolism of Berry Anthocyanins to Phenolic Acids in Humans. Journal of Agricultural and Food Chemistry, 2009. 57(6): p. 2274-2281. |
| 1. Paquette, M., et al., Strawberry and cranberry polyphenols improve insulin sensitivity in insulin-resistant, non-diabetic adults: a parallel, double-blind, controlled and randomised clinical trial. British Journal of Nutrition, 2017. 117(4): p. 519-531. |
| 1. Rebello, C.J., et al., Gastrointestinal microbiome modulator improves glucose tolerance in overweight and obese subjects: A randomized controlled pilot trial. Journal of Diabetes and Its Complications, 2015. 29(8): p. 1272-1276. |
| 1. Sakakibara, H., et al., Practical application of flavonoid-poor menu meals to the study of the bioavailability of bilberry anthocyanins in human subjects. Biosci Biotechnol Biochem, 2014. 78(10): p. 1748-52. |
| 1. Seymour, E.M., et al., Anthocyanin pharmacokinetics and dose-dependent plasma antioxidant pharmacodynamics following whole tart cherry intake in healthy humans. Journal of Functional Foods, 2014. 11: p. 509-516. |
| 1. Tarola, A.M., et al., Determination of Phenolic Compounds in Strawberries (Fragaria ananassa Duch) by High Performance Liquid Chromatography with Diode Array Detection. Food Analytical Methods, 2013. 6(1): p. 227-237. |
| 1. Williamson, G. and M.N. Clifford, Colonic metabolites of berry polyphenols: the missing link to biological activity? British Journal of Nutrition, 2010. 104: p. S48-S66. |
| 1. Yao, L.J., et al., Highly Selective Separation and Purification of Anthocyanins from Bilberry Based on a Macroporous Polymeric Adsorbent. Journal of Agricultural and Food Chemistry, 2015. 63(13): p. 3543-3550. |
| 1. Badjakov, I., et al., Bioactive compounds in small fruits and their influence on human health. Biotechnology & Biotechnological Equipment |
