# Supplementary material for: Contribution of Berry Polyphenols to the Human Metabolome
Source: Molecules. 2019 Nov 20;24(23):4220. doi: 10.3390/molecules24234220 (PMC6930569; doi:10.3390/molecules24234220)
Supplement: Supplementary file 1 [file molecules-24-04220-s001.zip › Supplement 6.docx]

**S6. Included manuscripts - reference list**

| 1. Aaby, K., et al., Phenolic compounds in strawberry (Fragaria x ananassa Duch.) fruits: Composition in 27 cultivars and changes during ripening |
| --- |
| 1. Alvarez-Fernandez, M.A., et al., Effects of the strawberry (Fragaria ananassa) puree elaboration process on non-anthocyanin phenolic composition and antioxidant activity |
| 1. Ariza, M.T., et al., Bioaccessibility and potential bioavailability of phenolic compounds from achenes as a new target for strawberry breeding programs |
| 1. Azzini, E., et al., Bioavailability of strawberry antioxidants in human subjects. |
| 1. Carkeet, C., B.A. Clevidence, and J.A. Novotny, Anthocyanin excretion by humans increases linearly with increasing strawberry dose |
| 1. Cerda, B., F.A. Tomas-Barberan, and J.C. Espin, Metabolism of antioxidant and chemopreventive ellagitannins from strawberries, raspberries, walnuts, and oak-aged wine in humans: Identification of biomarkers and individual variability |
| 1. Edirisinghe, I., et al., Strawberry anthocyanin and its association with postprandial inflammation and insulin. |
| 1. Felgines, C., et al., Strawberry anthocyanins are recovered in urine as glucuro- and sulfoconjugates in humans. |
| 1. Fernandes, I., et al., Antioxidant and antiproliferative properties of methylated metabolites of anthocyanins |
| 1. Holzwarth, M., et al., Evaluation of the effects of different freezing and thawing methods on color, polyphenol and ascorbic acid retention in strawberries (Fragaria x ananassa Duch.). Food Research International |
| 1. Ibero-Baraibar, I., et al., Different postprandial acute response in healthy subjects to three strawberry jams varying in carbohydrate and antioxidant content: |
| 1. Kalt, W., et al., Anthocyanin Metabolites Are Abundant and Persistent in Human Urine |
| 1. Kalt, W., et al., Flavonoid Metabolites in Human Urine during Blueberry Anthocyanin Intake. |
| 1. Kalt, W., et al., Human anthocyanin bioavailability: effect of intake duration and dosing |
| 1. Karlund, A., et al., Metabolic profiling discriminates between strawberry |
| 1. Ko, M.J., et al., Evaluation of Bioactive Compounds in Strawberry Fruits by a Targeted Metabolomic Approach |
| 1. Koli, R., et al., Bioavailability of various polyphenols from a diet containing moderate amounts of berries. |
| 1. Krikorian, R., et al., Blueberry Supplementation Improves Memory in Older Adults. |
| 1. Kuntz, S., et al., Anthocyanins from fruit juices improve the antioxidant status of healthy young female volunteers without affecting anti-inflammatory parameters: results from the randomised, double-blind, placebo-controlled, cross-over ANTHONIA (ANTHOcyanins in Nutrition Investigation Alliance) study. Br J Nutr, 2014. 112(6): p. 925-36. |
| 1. La Barbera, G., et al., Comprehensive polyphenol profiling of a strawberry extract (Fragaria x ananassa) by ultra-high-performance liquid chromatography coupled with high-resolution mass spectrometry. Analytical and Bioanalytical Chemistry, 2017. 409(8): p. 2127-2142. |
| 1. Lee, S., et al., Correlation between Species-Specific Metabolite Profiles and Bioactivities of Blueberries (Vaccinium spp.). Journal of Agricultural and Food Chemistry, 2014. 62(9): p. 2126-2133. |
| 1. Lowenthal, M.S., et al., Developing qualitative LC-MS methods for characterization of Vaccinium berry Standard Reference Materials. Analytical and Bioanalytical Chemistry, 2013. 405(13): p. 4451-4465. |
| 1. Ma, C.H., et al., Antioxidant and Metabolite Profiling of North American and Neotropical Blueberries Using LC-TOF-MS and Multivariate Analyses. Journal of Agricultural and Food Chemistry, 2013. 61(14): p. 3548-3559. |
| 1. Mazza, G., et al., Absorption of anthocyanins from blueberries and serum antioxidant status in human subjects. J Agric Food Chem, 2002. 50(26): p. 7731-7. |
| 1. McGhie, T.K., et al., Anthocyanin glycosides from berry fruit are absorbed and excreted unmetabolized by both humans and rats. J Agric Food Chem, 2003. 51(16): p. 4539-48. |
| 1. Milivojevic, J., et al., Classification and fingerprinting of different berries based on biochemical profiling and antioxidant capacity. Pesquisa Agropecuaria Brasileira, 2013. 48(9): p. 1285-1294. |
| 1. Misran, A., et al., Composition of phenolics and volatiles in strawberry cultivars and influence of preharvest hexanal treatment on their profiles. Canadian Journal of Plant Science, 2015. 95(1): p. 115-126. |
| 1. Mueller, D., et al., Human intervention study to investigate the intestinal accessibility and bioavailability of anthocyanins from bilberries. Food Chemistry, 2017. 231: p. 275-286. |
| 1. Mullen, W., et al., Bioavailability of pelargonidin-3-O-glucoside and its metabolites in humans following the ingestion of strawberries with and without cream. J Agric Food Chem, 2008. 56(3): p. 713-9. |
| 1. Mullen, W., et al., Use of accurate mass full scan mass spectrometry for the analysis of anthocyanins in berries and berry-fed tissues. J Agric Food Chem, 2010. 58(7): p. 3910-5. |
| 1. Nachar, A., et al., Phenolic compounds isolated from fermented blueberry juice decrease hepatocellular glucose output and enhance muscle glucose uptake in cultured murine and human cells. Bmc Complementary and Alternative Medicine, 2017. 17: p. 10. |
| 1. Nieman, D.C., et al., Influence of a polyphenol-enriched protein powder on exercise-induced inflammation and oxidative stress in athletes: a randomized trial using a metabolomics approach. PLoS One, 2013. 8(8): p. e72215. |
| 1. Nilsson, A., et al., Effects of a mixed berry beverage on cognitive functions and cardiometabolic risk markers; A randomized cross-over study in healthy older adults. Plos One, 2017. 12(11): p. 22. |
| 1. Nyambe-Silavwe, H. and G. Williamson, Polyphenol- and fibre-rich dried fruits with green tea attenuate starch-derived postprandial blood glucose and insulin: a randomised, controlled, single-blind, cross-over intervention. Br J Nutr, 2016. 116(3): p. 443-50. |
| 1. Overall, J., et al., Metabolic Effects of Berries with Structurally Diverse Anthocyanins. Int J Mol Sci, 2017. 18(2). |
| 1. Park, E., et al., A dose-response evaluation of freeze-dried strawberries independent of fiber content on metabolic indices in abdominally obese individuals with insulin resistance in a randomized, single-blinded, diet-controlled crossover trial. Mol Nutr Food Res, 2016. 60(5): p. 1099-109. |
| 1. Parra-Palma, C., et al., Linking the platelet antiaggregation effect of different strawberries species with antioxidants: Metabolomic and transcript profiling of polyphenols. Boletin Latinoamericano Y Del Caribe De Plantas Medicinales Y Aromaticas, 2018. 17(1): p. 36-52. |
| 1. Pedersen, C.B., et al., Effects of blueberry and cranberry juice consumption on the plasma antioxidant capacity of healthy female volunteers. Eur J Clin Nutr, 2000. 54(5): p. 405-8. |
| 1. Prencipe, F.P., et al., Metabolite profiling of polyphenols in Vaccinium berries and determination of their chemopreventive properties. Journal of Pharmaceutical and Biomedical Analysis, 2014. 89: p. 257-267. |
| 1. Prymont-Przyminska, A., et al., Consumption of strawberries on a daily basis increases the non-urate 2,2-diphenyl-1-picryl-hydrazyl (DPPH) radical scavenging activity of fasting plasma in healthy subjects. Journal of Clinical Biochemistry and Nutrition, 2014. 55(1): p. 48-55. |
| 1. Puupponen-Pimia, R., et al., Effects of ellagitannin-rich berries on blood lipids, gut microbiota, and urolithin production in human subjects with symptoms of metabolic syndrome. Molecular Nutrition & Food Research, 2013. 57(12): p. 2258-2263. |
| 1. Ramirez, J.E., et al., Anthocyanins and antioxidant capacities of six Chilean berries by HPLC-HR-ESI-ToF-MS. Food Chem, 2015. 176: p. 106-14. |
| 1. Ribnicky, D.M., et al., Effects of a high fat meal matrix and protein complexation on the bioaccessibility of blueberry anthocyanins using the TNO gastrointestinal model (TIM-1). Food Chemistry, 2014. 142: p. 349-357. |
| 1. Richter, C.K., et al., Incorporating freeze-dried strawberry powder into a high-fat meal does not alter postprandial vascular function or blood markers of cardiovascular disease risk: a randomized controlled trial. Am J Clin Nutr, 2017. 105(2): p. 313-322. |
| 1. Rodriguez-Mateos, A., et al., Bioavailability of wild blueberry (poly)phenols at different levels of intake. Journal of Berry Research, 2016. 6(2): p. 137-148. |
| 1. Rodriguez-Mateos, A., et al., Impact of processing on the bioavailability and vascular effects of blueberry (poly)phenols. Mol Nutr Food Res, 2014. 58(10): p. 1952-61. |
| 1. Rodriguez-Mateos, A., et al., Intake and time dependence of blueberry flavonoid-induced improvements in vascular function: a randomized, controlled, double-blind, crossover intervention study with mechanistic insights into biological activity. American Journal of Clinical Nutrition, 2013. 98(5): p. 1179-1191. |
| 1. Russell, W.R., et al., Availability of blueberry phenolics for microbial metabolism in the colon and the potential inflammatory implications. Mol Nutr Food Res, 2007. 51(6): p. 726-31. |
| 1. Sandhu, A.K., et al., Metabolic fate of strawberry polyphenols after chronic intake in healthy older adults. Food & Function, 2018. 9(1): p. 96-106. |
| 1. Sandhu, A.K., et al., Pharmacokinetic Characterization and Bioavailability of Strawberry Anthocyanins Relative to Meal Intake. J Agric Food Chem, 2016. 64(24): p. 4891-9. |
| 1. Schell, J., et al., Strawberries Improve Pain and Inflammation in Obese Adults with Radiographic Evidence of Knee Osteoarthritis. Nutrients, 2017. 9(9): p. 13. |
| 1. Seeram, N.P., et al., Cyclooxygenase inhibitory and antioxidant cyanidin glycosides in cherries and berries. Phytomedicine, 2001. 8(5): p. 362-9. |
| 1. Seeram, N.P., et al., Identification of phenolic compounds in strawberries by liquid chromatography electrospray ionization mass spectroscopy. Food Chemistry, 2006. 97(1): p. 1-11. |
| 1. Sharma, M., et al., Effects of Fruit Ellagitannin Extracts, Ellagic Acid, and Their Colonic Metabolite, Urolithin A, on Wnt Signaling. Journal of Agricultural and Food Chemistry, 2010. 58(7): p. 3965-3969. |
| 1. Song, J., et al., Quantitative changes in proteins responsible for flavonoid and anthocyanin biosynthesis in strawberry fruit at different ripening stages: A targeted quantitative proteomic investigation employing multiple reaction monitoring. Journal of Proteomics, 2015. 122: p. 1-10. |
| 1. Tonutare, T., U. Moor, and L. Szajdak, STRAWBERRY ANTHOCYANIN DETERMINATION BY pH DIFFERENTIAL SPECTROSCOPIC METHOD - HOW TO GET TRUE RESULTS? Acta Scientiarum Polonorum-Hortorum Cultus, 2014. 13(3): p. 35-47. |
| 1. Tulipani, S., et al., Antioxidants, phenolic compounds, and nutritional quality of different strawberry genotypes. Journal of Agricultural and Food Chemistry, 2008. 56(3): p. 696-704. |
| 1. Wu, X.L., et al., Concentrations of anthocyanins in common foods in the United States and estimation of normal consumption. Journal of Agricultural and Food Chemistry, 2006. 54(11): p. 4069-4075. |
| 1. Wu, X.L., G.H. Cao, and R.L. Prior, Absorption and metabolism of anthocyanins in elderly women after consumption of elderberry or blueberry. Journal of Nutrition, 2002. 132(7): p. 1865-1871. |
| 1. Xu, Y., et al., An effective method for preparation of high-purity pelargonidin-3-O-glucoside from strawberry and its protective effect on cellular oxidative stress. J Chromatogr B Analyt Technol Biomed Life Sci, 2018. 1072: p. 211-220. |
| 1. Yousef, G.G., et al., Efficient Quantification of the Health-Relevant Anthocyanin and Phenolic Acid Profiles in Commercial Cultivars and Breeding Selections of Blueberries (Vaccinium spp.). Journal of Agricultural and Food Chemistry, 2013. 61(20): p. 4806-4815. |
| 1. Yousef, G.G., et al., Impact of Interspecific Introgression on Anthocyanin Profiles of Southern Highbush Blueberry. Journal of the American Society for Horticultural Science, 2014. 139(2): p. 99-112. |
| 1. Zhang, J.J., et al., Metabolic profiling of strawberry (Fragariaxananassa Duch.) during fruit development and maturation. Journal of Experimental Botany, 2011. 62(3): p. 1103-1118. |
| 1. Zhang, M.L., J.H. Sun, and P. Chen, Development of a Comprehensive Flavonoid Analysis Computational Tool for Ultrahigh-Performance Liquid Chromatography-Diode Array Detection-High-Resolution Accurate Mass-Mass Spectrometry Data. Analytical Chemistry, 2017. 89(14): p. 7388-7397. |
| 1. Zheng, W. and S.Y. Wang, Oxygen radical absorbing capacity of phenolics in blueberries, cranberries, chokeberries, and lingonberries. J Agric Food Chem, 2003. 51(2): p. 502-9. |
| 1. Zhong, S.Q., et al., Characterization of Wild Blueberry Polyphenols Bioavailability and Kinetic Profile in Plasma over 24-h Period in Human Subjects. Molecular Nutrition & Food Research, 2017. 61(12): p. 13. |
