# Supplementary figures and images for: Contribution of Berry Polyphenols to the Human Metabolome
Source: Molecules. 2019 Nov 20;24(23):4220. doi: 10.3390/molecules24234220 (PMC6930569; doi:10.3390/molecules24234220)

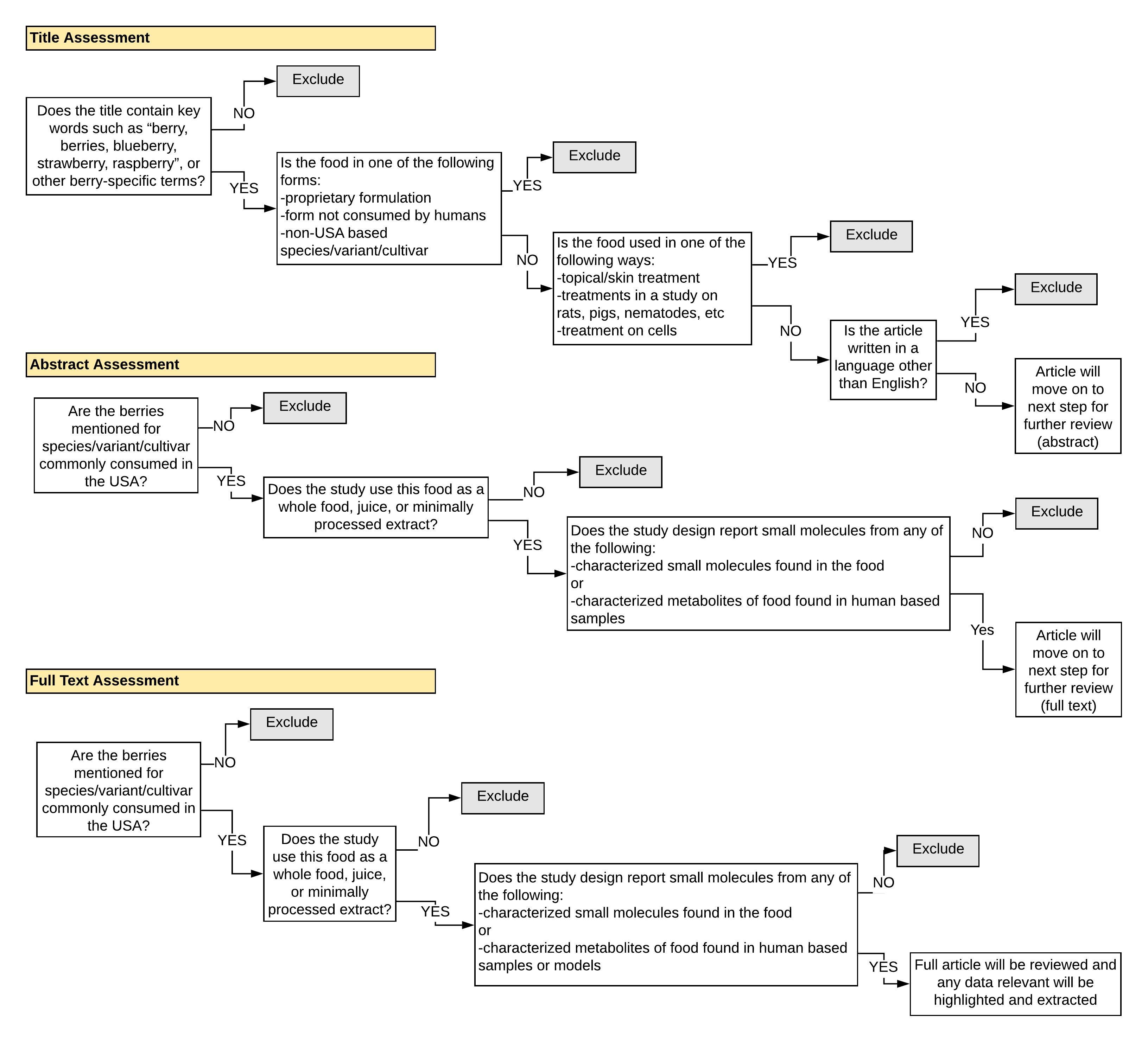

Supplement: Supplementary file 1 [file molecules-24-04220-s001.zip › Supplement 1 Figure1.jpeg]
